# The Influence of Key Facial Features on Recognition of Emotion in Cartoon Faces

**DOI:** 10.3389/fpsyg.2021.687974

**Published:** 2021-08-10

**Authors:** Shu Zhang, Xinge Liu, Xuan Yang, Yezhi Shu, Niqi Liu, Dan Zhang, Yong-Jin Liu

**Affiliations:** ^1^Department of Computer Science and Technology, Tsinghua University, Beijing, China; ^2^Beijing National Research Center for Information Science and Technology, Beijing, China; ^3^Department of Psychology, Tsinghua University, Beijing, China; ^4^Tsinghua Laboratory of Brain and Intelligence, Tsinghua University, Beijing, China; ^5^Key Laboratory of Pervasive Computing, Ministry of Education, Beijing, China

**Keywords:** cartoon faces, emotion recognition, facial features, expression intensity, happy, sad

## Abstract

Cartoon faces are widely used in social media, animation production, and social robots because of their attractive ability to convey different emotional information. Despite their popular applications, the mechanisms of recognizing emotional expressions in cartoon faces are still unclear. Therefore, three experiments were conducted in this study to systematically explore a recognition process for emotional cartoon expressions (happy, sad, and neutral) and to examine the influence of key facial features (mouth, eyes, and eyebrows) on emotion recognition. Across the experiments, three presentation conditions were employed: (1) a full face; (2) individual feature only (with two other features concealed); and (3) one feature concealed with two other features presented. The cartoon face images used in this study were converted from a set of real faces acted by Chinese posers, and the observers were Chinese. The results show that happy cartoon expressions were recognized more accurately than neutral and sad expressions, which was consistent with the happiness recognition advantage revealed in real face studies. Compared with real facial expressions, sad cartoon expressions were perceived as sadder, and happy cartoon expressions were perceived as less happy, regardless of whether full-face or single facial features were viewed. For cartoon faces, the mouth was demonstrated to be a feature that is sufficient and necessary for the recognition of happiness, and the eyebrows were sufficient and necessary for the recognition of sadness. This study helps to clarify the perception mechanism underlying emotion recognition in cartoon faces and sheds some light on directions for future research on intelligent human-computer interactions.

## Introduction

As an attractive art form, cartoon faces are widely used in daily life. Cartoon animation is an important carrier that not only helps children acquire emotional knowledge (Baron-Cohen et al., 2009; Schlosser et al., 2019) but enables adults to express feelings and attitudes (Jonassaint et al., 2018). In the field of artificial intelligence and human-robot interaction research, there has also been an urgent demand to incorporate emotional and sociable cartoon characters into the development of intelligent robots and virtual agents (Azevedo et al., 2019; Jaiswal et al., 2020). These non-realistic agents with emotionally expressive, human-like cartoon faces will be treated as partners instead of tools (Breazeal, 2003) and are widely used in a variety of applications, namely, education, entertainment, and healthcare (Breazeal, 2003). Although sophisticated computing models have been developed to animate emotional facial expressions with different types of artistic cartoon avatars in 2D and 3D (Zaharia et al., 2008; Obaid et al., 2010; Liu et al., 2013; Yu et al., 2015), some critical issues remain unclear, such as how people recognize emotional facial expressions in cartoon faces and underlying perception mechanisms. A better understanding of emotional facial expression recognition in cartoon faces would provide not only a theoretical reference for human-intelligence interaction but also emotional information for the development of emotionally expressive cartoon characters for artificial intelligence and sociable robot applications.

Cartoons are a kind of illustration with different styles from ridiculous exaggerating characteristics in caricature (Benson and Perrett, 1991). Cartoons typically have a non-realistic or semi-realistic style and draw from a common canon of iconic facial expression illustrations to denote particular moods and thoughts (Wikipedia, 2021). In recent years, Japanese and American cartoons have been dominant in popular culture. Generally, cartoon faces have non-realistic facial features. For example, some researchers systematically examined the size difference in facial features between real human faces and faces in different animation genres and found that the eyes, nose, ears, forehead, and chin tended to be exaggerated in both American and Japanese cartoon characters (Liu et al., 2019). In addition, cartoon faces maintain low-level metric parameters and face proportions but lack high-level information on human faces, such as skin texture, skeletal structure, and anatomic structures. Compared with other kinds of non-realistic images of faces, cartoon faces are not as highly simplified as schematic and iconic faces, which represent facial expressions with a minimal number of pencil strokes (Fujiwara et al., 2002; Breazeal, 2003), or as highly realistic as caricatures and portraits. Many computational models have been developed to automatically transform real faces into artistically stylized cartoon faces; however, these algorithms are challenged by the use of parametric techniques and physical models that generate facial expressions by exaggerating the size of facial features (eyes, eyebrows, lips, and mouth) and deforming their shapes (Zaharia et al., 2008).

Emotional expression is a kind of facial information. Although research has found that emotional facial expression recognition can become more accurate and effective as facial stimuli become more abstract (Kendall et al., 2016), little is known about the influence of exaggerated and stylized facial features in cartoon faces on emotional expression recognition. Studies using high-level simplified non-real faces, such as emoticons and stick figures, supported the view that emotions are recognized more quickly with these cartoon faces than with real faces (Ikeda, 2020; Wessler and Hansen, 2021). Furthermore, the difference in the holistic processing of emotional expression when real and non-real faces are used may imply that faces with exaggerated and stylized facial features are less holistically processed when perceiving expression. For example, Prazak and Burgund (2014) used a composite facial expression recognition task in which half of the happy faces and half of the sad faces were combined into a composite face, and the participants were asked to identify the facial expression based on the emotional expression of the upper half of the face; they found greater holistic processing for real faces than for schematic faces. Considering that facial images in Japanese cartoons have more exaggerated features than those in American cartoons (Liu et al., 2019), we used Japanese cartoon faces as facial stimuli and aimed to explore the contribution of facial features to the recognition of emotional expression in cartoon faces.

Despite the increasing popularity of cartoon faces, investigations of the mechanisms underlying emotion recognition remain limited. However, there is a large number of studies on the recognition of emotional expressions in real faces that could inspire studies with cartoon faces. Regarding emotion recognition, previous results have shown that emotional expressions are not equally processed accurately. Happiness holds a recognition advantage over other emotional expressions; that is, happy expressions are identified more accurately with less cognitive effort (Kirita and Endo, 1995; Leppänen and Hietanen, 2004; Du and Martinez, 2013; Nummenmaa and Calvo, 2015). Regarding the perception mechanism, two models have been proposed such as the feature model and the holistic model (Calvo and Nummenmaa, 2016).

The feature model refers to a part-based process of expression recognition and suggests that the success of emotional expression recognition could be dependent on specific single facial features (Beaudry et al., 2014; Tobin et al., 2016). Unlike the holistic model, each component of facial stimulus can express emotion out of context (for a review see Bimler and Paramei, 2006). On the other hand, the holistic model argues that a single facial feature is not sufficient to identify the target emotional expression and posits that successful expression recognition is based on the whole facial configuration (Piepers and Robbins, 2012). Evidence from the last two decades has revealed a complex picture of recognition of emotional expression on real human faces (Calder and Jansen, 2005; Calvo and Nummenmaa, 2008; Nusseck et al., 2008; Tanaka et al., 2012; Beaudry et al., 2014). First, facial emotional expression recognition varies as a function of emotion. For example, Calvo and Nummenmaa (2008) found that happy, surprised, and disgusted expressions rely more on featural information, while fearful, angry, and sad expressions rely more on holistic processing. Second, single facial features have been identified as sufficient or necessary to discriminate some facial expressions. Specifically, the mouth has been consistently found to be both sufficient and necessary to discriminate happiness (Calder and Jansen, 2005; Nusseck et al., 2008; Bombari et al., 2013; Beaudry et al., 2014; Maher et al., 2014). A smiling mouth has been widely considered as a salient and distinctive facial feature in previous studies (Calvo and Nummenmaa, 2016; Guarnera et al., 2017; Wegrzyn et al., 2017; Calvo et al., 2018), a finding that supports the feature-based process of happiness recognition (Beaudry et al., 2014). The eye region, which includes the eyes and eyebrows, has been identified as important, sufficient, and necessary for discriminating sad expressions (Calvo et al., 2006; Calvo and Nummenmaa, 2008; Wegrzyn et al., 2017; Ikeda, 2020), but the eyes and eyebrows are seldom explored separately. It remains unclear whether the eyes and eyebrows play unique roles in this cognitive process (Beaudry et al., 2014).

Few studies have investigated the process of recognizing facial expressions in cartoon faces, and the results have been inconsistent. Some results support a happiness advantage similar to that for real faces (Kirita and Endo, 1995; Leppänen and Hietanen, 2004), whereas others reveal an anger or threat advantage (Lundqvist and Öhman, 2005; Calvo et al., 2006; Lipp et al., 2009) conducted a facial inverse (upside down face) task with both schematic and real faces and found that the response time was not significantly different between upright and inverted faces, suggesting a similar feature-based process for non-real and real faces. In contrast, Rosset et al. (2008) found that a facial inversion effect with cartoon faces existed for both typically developing children and children with autism, e.g., inverting cartoon faces decreased the ability of children with autism to identify their expression, suggesting that cartoon faces are holistically processed.

Regarding the contribution of single facial features, some preliminary results have revealed that the recognition of different expressions of emotions relies on different types of information (Bimler and Paramei, 2006; Smith and Schyns, 2009; Koda et al., 2011; Bombari et al., 2013; Du and Martinez, 2013; Maher et al., 2014; Rossion, 2014; Calvo and Nummenmaa, 2016; Calvo et al., 2018) found that the mouth is a key feature in the detection of happiness and fear and that the region of the eyes is more important in the detection of anger, fear, and sadness. However, it remains unknown whether the exaggerated and stylized single features in cartoon faces are sufficient or necessary to effectively convey the same degree of emotional information that they do in real faces. In cartoon faces, some facial features, such as eyes, are exaggerated in size, and some features are artistically stylized, such as the exaggerated and distinct smile of a clown; therefore, they may attract attention more easily and facilitate the perception process. These exaggerated features are typical and might be sufficient for effective expression recognition, which suggests the occurrence of feature-based processing. To this end, based on inconsistent findings and a paucity of research, further exploration of the mechanism underlying the perception of emotional facial expressions in cartoon faces is required.

Although previous studies have used abstract non-real faces with facial configurations similar to those of real faces (Lundqvist and Öhman, 2005; Calvo et al., 2006; Rosset et al., 2008; Kendall et al., 2016), some critical questions remain, such as whether cartoon facial expressions convey a higher degree of emotional intensity than real ones and what the mechanism is for perceiving cartoon facial expression. Therefore, in this study, three experiments were conducted to explore the recognition of emotional facial expressions in cartoon faces and the contribution of single features. Experiment 1 aimed to explore whether the accuracy and intensity perception of emotional expressions differ for cartoon faces and real human faces when whole faces are presented. Experiments 2 and 3 were conducted to examine the sufficiency and necessity of single facial features for emotional expression recognition. To verify the potential feature-based model, the widely used hidden-or-presented expression recognition paradigm (Beaudry et al., 2014) was applied to assess the sufficiency and necessity of single facial features for the detection of emotional expressions. Three representative emotions (happy, neutral, and sad) and three facial features (eyes, eyebrows, and mouth) were included in this study. A careful examination of whether and how recognition of facial emotional expressions in cartoon faces resemble and differ from recognition of the emotional expressions of real human faces not only would help clarify the mechanism of expression recognition but would also provide a theoretical foundation for artificial intelligence research, such as the development of computational models and human-robot interactions.

## Experiment 1: Recognition of Emotional Information on Cartoon Faces and Real Faces

The main objective of Experiment 1 was to investigate the characteristics of emotional expression recognition for cartoon faces. Since the features of cartoon faces are exaggerated or simplified, making it easier to discriminate their facial expressions (Kendall et al., 2016; Ikeda, 2020; Wessler and Hansen, 2021), we hypothesized that (1) emotion recognition accuracy would be higher for cartoon faces than for real faces and (2) the two types of faces would convey different levels of emotional intensity. Because of the lack of research investigating the difference in emotional intensity perception of cartoon facial expressions, specific hypotheses were not proposed regarding perceived intensity.

### Participants

The sample sizes for the experiments in this study were based on exploratory parameter estimation using the power analysis calculator at https://jakewestfall.shinyapps.io/pangea/ (Judd et al., 2017) with a moderate effect size of *d* = 0.45 (Lachenbruch, 1989), α = 0.05, and power > 0.8. All the experiments were approved by the Institutional Review Board of the Department of Psychology at Tsinghua University and were conducted according to the ethical standards stipulated in the 1964 Declaration of Helsinki. The participants were recruited from Tsinghua University, and they all agreed and signed informed consent before participating in the experiment; they earned an appropriate reward for completing it.

Exclusion criteria for participation included but were not limited to (1) any mental disorders, brain trauma, or trauma; (2) cold symptoms and neurologic drug intake within a week immediately before the experiment; and (3) a Self-Rating Depression Scale (SDS) score > 24 (Zung, 1965). Self-reported adequate sleep with no consumption of drinks or medicine containing alcohol, caffeine, or other excitatory substances for at least 24 h before the experiment was required for this study.

The estimated sample size of Experiment 1 was 30 with a statistical power of 0.82. We recruited 30 Chinese participants (female/male: 16/14; mean age ± SD = 22.57 ± 2.56 years old). All of them were right-handed with normal or corrected-to-normal vision.

### Stimuli

Previous studies have demonstrated that facial recognition is sensitive to differences in the race (Ekman and Friesen, 1971; Ekman et al., 1987; Ng and Lindsay, 1994); therefore, to exclude cross-race influences on facial expression recognition, facial emotional expression images were selected from the Tsinghua facial expression database, which is a database of facial expressions posed by young and older Chinese women and men (Yang et al., 2020). In this study, we selected happy, neutral, and sad expressions with correct categorization rates of 97.77, 84.97, and 76.41%, respectively (Yang et al., 2020). Since the algorithm (Kim et al., 2019) used for real-to-cartoon face conversion in this study was established using female images, all the facial expression images selected from the database were images of young female actors (20 in total). First, the human facial images were converted into a Japanese cartoon style using the U-GAT-IT computational model, and the effect size was computed by the following formula (Kim et al., 2019):

KID(kernelinceptiondistance)×100±std.×100=11.61±0.57.

**FIGURE 1:**
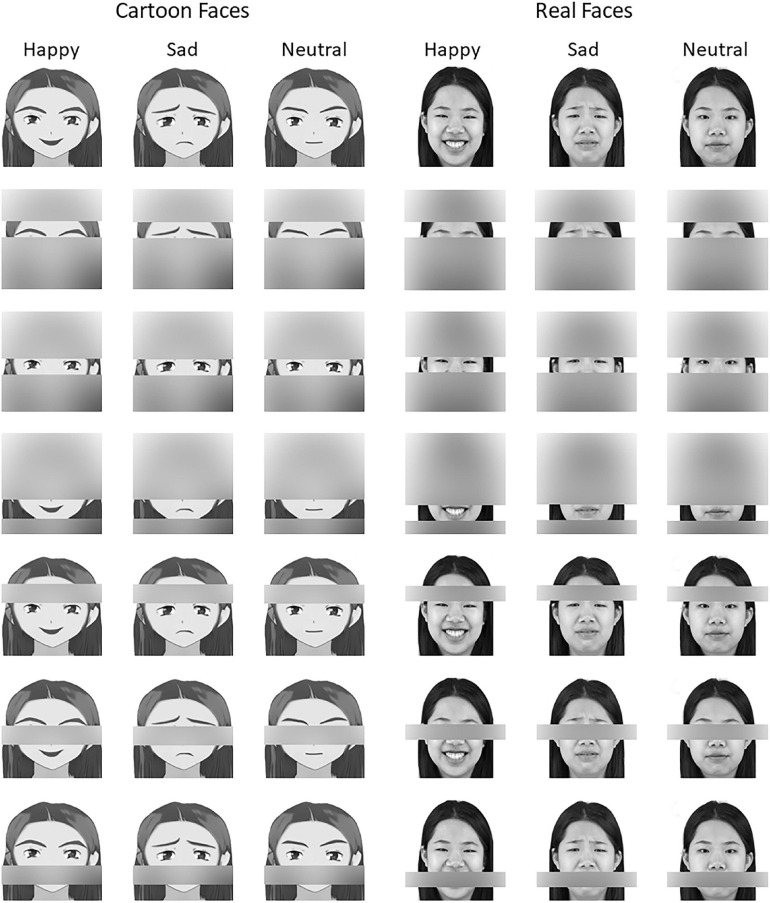
Examples of stimuli.

As facial emotional expression learning was absent when the U-GAT-IT computational model was trained (Kim et al., 2019), the quality of the emotional expressions of the cartoon images was reviewed and revised by an artist using Adobe Photoshop CS6 to ensure that their emotional facial expressions were consistent with the corresponding real expressions (as shown in Figure 1 for an illustration). After that, to verify whether the cartoon stimuli conveyed the target emotional expressions correctly, an additional 30 Tsinghua undergraduates were invited to label and evaluate these facial expressions (happy, neutral, or sad). The mean categorization accuracy was over 90% (cartoon: 95.5%, real: 94.61%; details of the pre-experimental procedure and statistical results are provided in Supplementary Part 1). Finally, 20 cartoon characters based on 20 real images of people with happy, sad, and neutral facial expressions were selected for this study. All the stimuli used in this study were converted to grayscale images and resized to 300 × 300 pixels.

### Design and Procedure

We used a 2 (type: cartoon vs. real) × 3 (expression: happy vs. neutral vs. sad) within-participants design and conducted all the experiments in the standard behavioral laboratory of the Psychology Department of Tsinghua University. The stimuli were presented against a gray background using the E-prime 2.0 software (RGB values: 128, 128, and 128) on a 23.8-inch monitor with a resolution of 1,920 × 1,080 pixels and a 60-Hz refresh rate. The participants sat in a room and were 60 ± 10 cm away from the screen.

**FIGURE 2:**
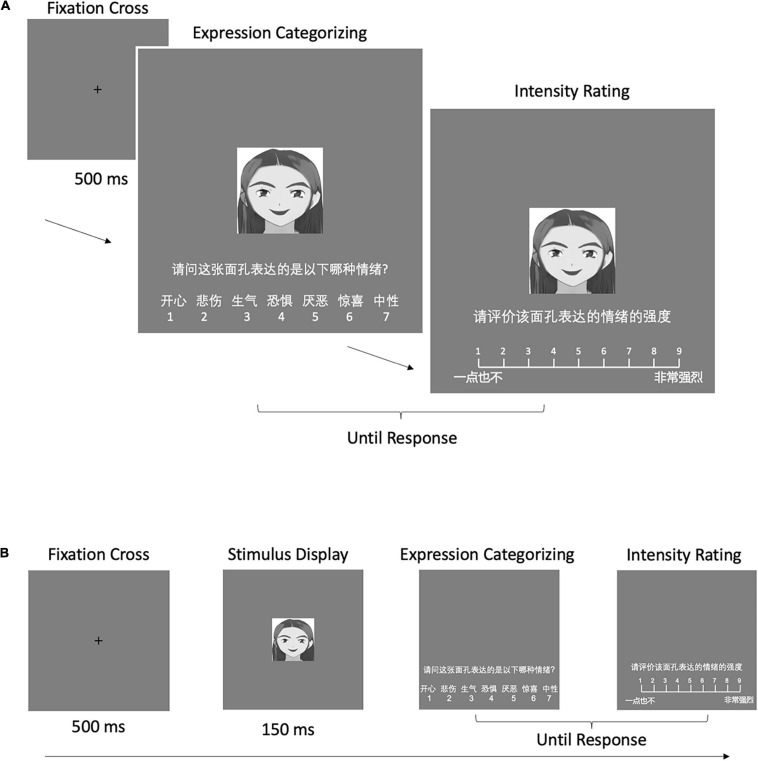
The sequence of a single trial in **(A)** Experiment 1 and **(B)** Experiments 2 and 3.

Each trial began with the presentation of a central fixation cross for 500 ms, and then a facial image appeared and remained until the participant responded. For the consideration of the six widely accepted basic emotions proposed by Ekman and Friesen (1978) and to prevent an accuracy ceiling effect for emotional recognition, the participants were asked to identify the presented emotional expression from seven emotional categories (happy, sad, angry, disgust, fear, surprise, and neutral) presented underneath the facial image. Subsequently, a 9-point scale of intensity (1 = “not intense at all,” 9 = “extremely intense”) was presented at the bottom center of the screen, and the participants were asked to rate the perceived emotional intensity of the same image. The order of the options was counterbalanced across the participants, who were required to identify the emotion of the facial images and rate their intensity by pressing the corresponding key as accurately and quickly as possible. The target facial expression image was presented until two responses were input. To become familiar with the tasks, the participants practiced a trial for each condition for a total of six trials. The facial images used in the practice session were not used in the main experiments. In the formal phase, cartoon and real faces were presented in different blocks in a counterbalanced order across the participants as with previous studies (Kendall et al., 2016; Zhao et al., 2019). The expressions of facial images were presented randomly within blocks. The participants completed 120 experimental trials with 20 facial images for each emotional expression. Examples of the sequence of a single trial used in the experiment are shown in Figure 2A.

### Analysis

The expression recognition accuracy (hereinafter called accuracy) was the number of correct responses divided by the total number of trials for each target emotion. The percentage of false responses was the ratio of the number of choosing each non-target emotional category to the total number of trials for the particular target emotion. To test whether neutral expressions are perceived with a “residual” affective meaning, one-sample *t*-tests were conducted to compare the percentages of false responses with the chance level accuracy (Russell and Fehr, 1987; Kesler et al., 2001; Lee et al., 2008; Azevedo et al., 2019). Refer to Supplementary Part 2 for detailed results of the false responses of Experiments 1, 2, and 3. Perceived expression intensity (hereinafter called intensity) was the average intensity rating of each target emotional expression. The original data of this study are available at https://github.com/Nicki-Liu/Emotion/tree/main/data. All statistical analyses were performed using SPSS 25.0 (IBM SPSS Statistics, New York). All contrasts were Bonferroni-corrected for multiple comparisons, and a *p*-value < 0.05 was considered statistically significant. A greenhouse-Geisser correction was applied when the sphericity hypothesis was violated.

### Results

**FIGURE 3:**
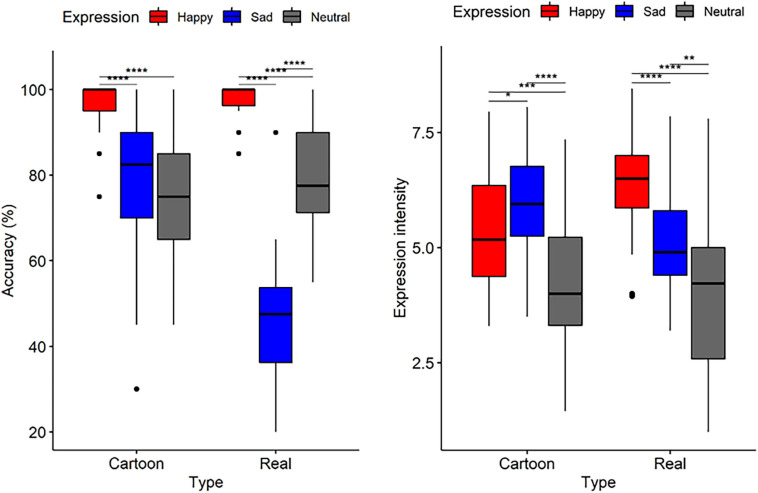
Boxplots for recognition accuracy **(left)** and perceived expression intensity **(right)** for judging cartoon and real-face expressions in Experiment 1. Here and henceforth stars indicate the following levels of significance: *p < 0.05, **p < 0.01, ***p < 0.001, and ****p < 0.0001, in multiple contrasts, after Bonferroni corrections.

Repeated measures ANOVAs (type: cartoon vs. real; expression: happy vs. neutral vs. sad) for accuracy (Figure 3, left) revealed significant main effects of type [*F*_(1, 29)_ = 0.26, *p* < 0.001, η_p_^2^ = 0.42] and expression [*F*_(2, 58)_ = 90.1, *p* < 0.001, η_p_^2^ = 0.75] and a significant interaction effect between them [*F*_(1__.52__, 43__.95__)_ = 47.84, *p* < 0.001, η_p_^2^ = 0.62]. The simple effects analysis showed that the accuracy of happiness recognition [*M* = 0.97, *SE* = 0.01, 95% CI = (0.95, 0.99)] was higher than that of neutral [*M* = 0.76, *SE* = 0.03, 95% CI = (0.7, 0.81)] and sad [*M* = 0.77, *SE* = 0.04, 95% CI = (0.69, 0.85)] recognition for cartoon faces, *p*s < 0.001. For real faces, the accuracy decreased from happy [*M* = 0.98, *SE* = 0.01, 95% CI = (0.96, 0.99)] to neutral [*M* = 0.79, *SE* = 0.02, 95% CI = (0.74, 0.84)] and then to sad expressions [*M* = 0.47, *SE* = 0.03, 95% CI = (0.41, 0.52)], *p*s < 0.001. Furthermore, only the sad expression showed higher accuracy for the cartoon faces than for the real faces (*p* < 0.001). No difference was observed for the happy and neutral expressions between the cartoon and real faces (*p*s > 0.19).

The results for intensity (Figure 3, right) showed a main effect of expression [*F*_(1__.44__, 41__.7__)_ = 33.77, *p* < 0.001, η_p_^2^ = 0.54]. The main effect of type was not significant [*F*_(1, 29)_ = 0.27, *p* = 0.605, η_p_^2^ = 0.009]. The interaction effect was significant [*F*_(2, 58)_ = 32.42, *p* < 0.001, η_p_^2^ = 0.53]. A simple main effect analysis showed that for the cartoon faces, the perceived intensity of sadness [*M* = 5.96, *SE* = 0.22, 95% CI = (5.51, 6.42)] was higher than that of happiness [*M* = 5.39, *SE* = 0.24, 95% CI = (4.89, 5.89)], and that the neutral expression [*M* = 4.21, *SE* = 0.29, 95% CI = (3.61, 4.81)] had the lowest perceived intensity, *p*s < 0.025. For the real faces, the intensity of happiness [*M* = 6.47, *SE* = 0.2, 95% CI = (6.07, 6.87)] was significantly higher than that of sadness [*M* = 5.18, *SE* = 0.22, 95% CI = (4.73, 5.63)], and the neutral expression [*M* = 4.09, *SE* = 0.33, 95% CI = (3.41, 4.77)] had the lowest perceived intensity, *p*s < 0.003. The interaction was decomposed in another direction to reveal the effect of types on the expressions. The results of a simple effects analysis showed that the perceived intensity of the happy expression was higher for the real faces than for the cartoon faces (*p* < 0.001), while the perceived intensity of the sad expression was higher for the cartoon faces than for the real faces (*p* < 0.001). No difference was found for the intensity of neutral expressions between the two face types (*p* = 0.523).

### Discussion

The main objective of Experiment 1 was to explore the recognition of emotional expressions in cartoon faces by comparing it with the recognition of emotional expressions in real faces. Two measures, recognition and perceived emotional intensity of the target facial expressions, were examined. The results reveal a happiness recognition advantage for the cartoon expressions that are similar to that for the real expressions. More importantly, significant differences were found between the responses to the cartoon and real faces. The accuracy of recognition and perceived emotional intensity of the target facial expressions varied as a function of face type and emotion category. The results show that the processing of emotion in cartoon faces share a similarity with the emotion processing mechanism in real faces, as the happy expression is identified more accurately than the neutral and sad expressions. This is in line with previous studies on real faces that found that the happy face advantage was a genuine psychological effect and that the recognition of happy faces has clear superiority over the recognition of other emotional expressions across all types of stimulus (Kirita and Endo, 1995; Leppänen and Hietanen, 2004; Nummenmaa and Calvo, 2015). It seems that the stylized and simplified cartoon facial expressions did not impair this happy advantage. However, it was inconsistent with the results of previous research using schematic faces (i.e., another type of non-real but more simplified faces), which failed to find a happy face advantage and even revealed a disadvantage (Lundqvist and Öhman, 2005; Calvo et al., 2006). The results reveal that the emotional information conveyed by cartoon facial expressions was not equivalent across emotion categories. Sad cartoon faces were recognized more precisely than sad real faces. A possible explanation for this difference may be affective uniqueness. In Experiment 1, in terms of the cartoon faces with sad expressions, the probability of a false response of other emotions was not greater than chance, suggesting that the sadness in cartoon facial expressions is recognized distinctly. In contrast, in terms of sad expressions in the real faces in this current study, the tendency to identify sad expressions as disgust [*M* = 0.28, SE = 0.04, 95% CI = (0.21, 0.36)], *p* < 0.001, refer Supplementary Part 2.1) was greater than chance, which may have impaired recognition accuracy. This explanation is consistent with previous studies that showed emotional expressions with a negative valence could be confused with one another in real faces (Palermo and Coltheart, 2004; Tottenham et al., 2009). Specifically, sadness could be confused with disgust (Tottenham et al., 2009), fear (Recio et al., 2013), and a neutral expression in real faces (Palermo and Coltheart, 2004; Tottenham et al., 2009). Bimler and Paramei (2006) found that configural information plays a crucial role in the decoding of emotions conveyed by facial expressions. Some curvatures were exaggerated in cartoon images, such as the elevated inward eyebrow parts (AU1) and the downward mouth curvature (AU24), which are less distinct in real images (Cohn et al., 2007). Further, due to the absence of nasolabial furrow, the landmark of the expression of disgust in cartoon faces, there was a great chance for cartoon sadness to be misidentified as disgust.

Regarding the perceived intensity of the target emotional expressions, differentiated results were found for different emotions and face types. The sad expressions in cartoon faces were perceived as sadder than those of real faces, and the happy expressions were perceived as less happy. According to previous studies, different facial features contribute differently to the identification of emotional expressions (Calvo and Nummenmaa, 2008; Nusseck et al., 2008; Beaudry et al., 2014).

Considering that some of the features of the cartoon faces were artificially exaggerated or simplified compared with those of the real faces, the different contributions of single facial features may have led to the differences across emotions between cartoon and real faces. For example, previous studies have shown that eyebrows, presented with clear and exaggerated pencil stroke lines, played a crucial role in the recognition of sadness (Hasegawa and Unuma, 2010).

## Experiment 2: Sufficiency of Specific Facial Features

The main objective of Experiment 2 was to investigate the sufficiency of specific facial features for the recognition of emotional expressions in cartoon faces. We referred to the approach used to manipulate facial feature stimuli in previous studies (Calvo and Nummenmaa, 2008; Beaudry et al., 2014). Specifically, a facial feature was considered sufficient if recognition accuracy, when only the feature was presented (e.g., mouth only), was not significantly different from recognition accuracy in the full-face condition. Because the features in cartoon faces are simplified but similar to those in real faces, we hypothesized that the sufficiency of facial features for the recognition of cartoon facial expressions should be similar to that for real faces, namely, the mouth region, which would be sufficient to identify happiness, and the eyes and eyebrows, which would be sufficient to identify sadness (Calder and Jansen, 2005; Calvo and Nummenmaa, 2008; Nusseck et al., 2008; Beaudry et al., 2014).

### Participants

Based on the exploratory parameter estimation described in Experiment 1, the estimated sample size for Experiment 2 was 21, with a statistical power of 0.81. We recruited 30 eligible Chinese students from Tsinghua University (female/male: 16/14; mean age ± SD = 21.93 ± 2.82 years old) to participate in this experiment. The exclusion and inclusion criteria were the same as those for Experiment 1.

### Stimuli

When presenting only a single feature, we concealed other facial features (present eyebrows, eyes, or mouth only) by Gaussian blur at an intensity of 60 (which guaranteed that concealed positions would not be recognized) coupled with a size of 60 × 256 pixels (height × width) without blur, which ensured that the target facial parts were completely presented. Aside from the Gaussian blur used to display the specific facial feature, all the stimuli and the apparatus were identical to those used in Experiment 1. Finally, 480 images (four cartoon and real features, three emotions, and 20 characters) were included in Experiment 2.

### Design and Procedure

Experiment 2 used a 2 (type: cartoon vs. real) × 3 (expression: happy vs. sad vs. neutral) × 4 (feature: mouth vs. eyes vs. eyebrows vs. full face) within-participant design. The procedure was identical to that of Experiment 1 except that in Experiment 2, the image was presented for 150 ms (Calvo and Beltrán, 2014), and that the presentation was not self-paced by the participants. The participants were required to identify the emotional expression and rate the intensity of the image presented. The order of presentation of mouths, eyes, and eyebrows was counterbalanced across blocks and participants. Finally, the full-face condition was presented to prevent the participants from becoming familiar with the facial stimuli. The order of presentation of cartoons and real faces was counterbalanced across participants. After six practice trials, 480 trials were presented in total (20 characters × 2 types × 3 expressions × 4 features).

### Results

We conducted 2 (type) × 3 (expression) × 4 (feature) repeated measures ANOVAs for accuracy and intensity separately. The three-way interaction (if it reached significance) was decomposed by splitting the type to specify the effect of features on cartoon emotional facial expression and by splitting the expression to specify the effect of types on feature expression recognition. For the sake of brevity, we focused on the results of the cartoon faces; other results of multiple comparisons are shown in the Supplementary Material.

#### Accuracy of Emotional Facial Expression Recognition

The results for accuracy revealed significant main effects of feature [*F*_(3, 87)_ = 170.02, *p* < 0.001, η_p_^2^ = 0.85] and expression [*F*_(1__.64__, 47__.43__)_ = 22.01, *p* < 0.001, η_p_^2^ = 0.43] and all interaction effects (*F*s > 28.53, *p*s < 0.001, η_p_^2^s > 0.5). The main effect of face type did not reach significance [*F*_(1, 29)_ = 0.84, *p* = 0.368, η_p_^2^ = 0.03].

**FIGURE 4:**
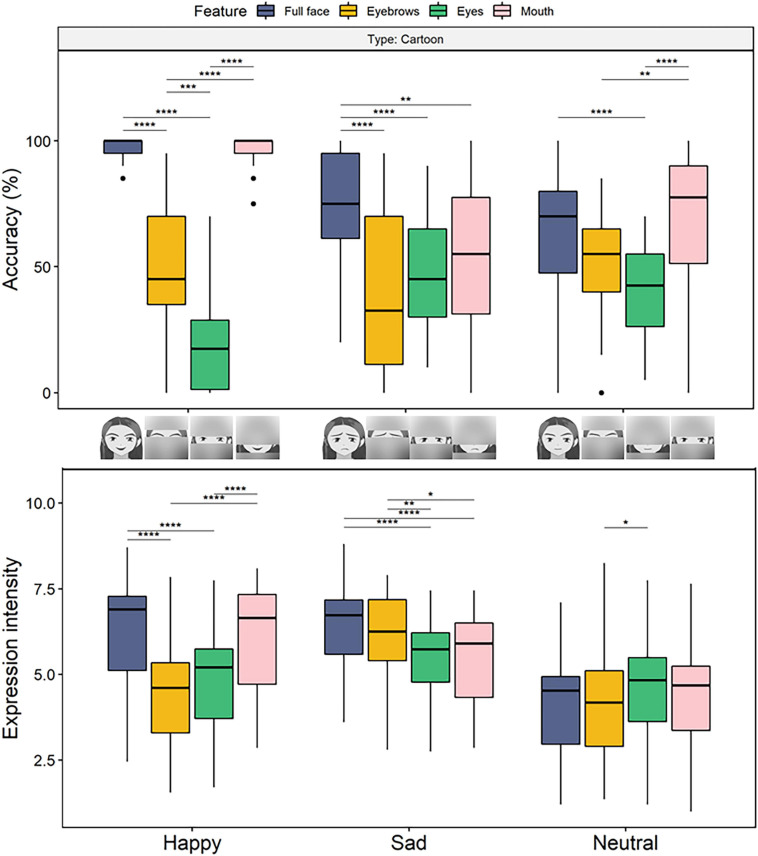
Boxplots for recognition accuracy **(top)** and perceived expression intensity **(bottom)** for the assessment of the expressions on cartoon faces as a function of facial features in Experiment 2. For happiness, presenting the mouth only had a little impact on the accuracy and intensity rating, while presenting the eyes and eyebrows only decreased the performance. For sadness, any feature that was presented only decreased the accuracy but presenting only the eyebrows did not affect the perception of sadness intensity. Here and henceforth stars indicate the following levels of significance: *p < 0.05, **p < 0.01, ***p < 0.001, and ****p < 0.0001, in multiple contrasts, after Bonferroni corrections.

To investigate the sufficiency of facial features in emotion recognition of cartoon faces, we decomposed a three-way interaction by conducting 3 (expression) × 4 (feature) repeated measures ANOVAs for different types. The results of the cartoon faces (Figure 4, top) revealed a main effect of feature and a significant interaction effect (*F*s > 22.6, *p*s < 0.001, η_p_^2^s > 0.43). For happiness recognition, the pairwise comparisons showed that the accuracy did not differ between the mouth-only [*M* = 0.96, *SE* = 0.01, 95% CI = (0.94, 0.99)] and full-face conditions [*M* = 0.97, *SE* = 0.01, 95% CI = (0.95, 0.98), *p* = 1], and both yielded greater accuracy than when only the eyebrows [*M* = 0.47, *SE* = 0.05, 95% CI = (0.37, 0.58)] or eyes [*M* = 0.2, *SE* = 0.04, 95% CI = (0.13, 0.28), *p*s < 0.001] were presented (*p*s < 0.001). For sadness recognition, the accuracy was higher for the full-face condition [*M* = 0.76, *SE* = 0.04, 95% CI = (0.68, 0.84)] than when any single facial feature was presented alone (*M*s < 0.56, *p*s < 0.013), and no differences were observed among the single features that were presented separately (*p*s > 0.243). Refer the Supplementary Part 3 for detailed results of the accuracy of the real faces.

We also decomposed three-way interactions by conducting 2 (type) × 4 (feature) repeated measures ANOVAs for different expressions (Supplementary Figure 2, top). The results of happiness recognition showed significant main effects of type, feature, and interaction effect (*F*s > 29.38, *p*s < 0.001, η_p_^2^s > 0.5), and pairwise comparisons revealed that the accuracy for the cartoon faces [*M* = 0.2, *SE* = 0.04, 95% CI = (0.13, 0.28)] was significantly lower than that for the real faces [*M* = 0.84, *SE* = 0.02, 95% CI = (0.8, 0.88)] when only the eyes were presented (*p* < 0.001) but was significantly higher for the cartoon faces [*M* = 0.47, *SE* = 0.05, 95% CI = (0.37, 0.58)] than for the real faces [*M* = 0.2, *SE* = 0.04, 95% CI = (0.13, 0.28)], when only the eyebrows were presented (*p* < 0.001). No significant difference between the two face types was observed when only the mouth (mean difference = 0.002) or the full face (mean difference = 0.008) was presented (*p*s > 0.475). For sadness recognition, two main effects and interaction reached significance, *F*s > 3.91, *p*s < 0.011, η_p_^2^s > 0.11). The accuracy of sadness recognition was higher for the cartoon faces than for the real faces across all conditions (*p*s < 0.029) with the differences between the two types increasing from the eyebrows (mean difference = 0.11, *SE* = 0.05) to eyes (mean difference = 0.17, *SE* = 0.04), the full face (mean difference = 0.17, *SE* = 0.4), and then the mouth (mean difference = 0.3, *SE* = 0.05).

#### Perceived Intensity of Emotional Facial Expressions

The results for intensity revealed main effects of feature [*F*_(3, 87)_ = 23.11, *p* < 0.001, η_p_^2^ = 0.44] and expression [*F*_(1__.42__, 42__.21__)_ = 68.44, *p* < 0.001, η_p_^2^ = 0.7] and effects of all the two-way and three-way interactions (*F*s > 9.36, *p*s < 0.001, η_p_^2^s > 0.24). The three-way interaction was decomposed in the same manner used in the analysis of recognition accuracy.

The results of the 3 × 4 repeated-measures ANOVAs for the cartoon faces (Figure 4, bottom) revealed significant main effects of feature and expression and an interaction effect of feature × expression (*F*s > 12.1, *p*s < 0.001, η_p_^2^s > 0.29). Furthermore, the pairwise comparisons revealed that the perceived intensity of happiness was significantly higher for the full-face [*M* = 6.21, *SE* = 0.28, 95% CI = (5.63, 6.78)] and mouth-only [*M* = 6.01, *SE* = 0.29, 95% CI = (5.41, 6.61)] conditions than for the eyes- [*M* = 4.7, *SE* = 0.27, 95% CI = (4.15, 5.26)] and eyebrows-only [*M* = 4.41, *SE* = 0.3, 95% CI = (3.79, 5.03)] conditions, *p*s < 0.001; no differences were observed between the full-face and mouth conditions or between the eyes and eyebrows conditions (*p*s > 0.931). For sad expressions, perceived intensity when the full-face [*M* = 6.44, *SE* = 0.23, 95% CI = (5.97, 6.92)] and eyebrows-only [*M* = 6.07, *SE* = 0.23, 95% CI = (5.59, 6.55)] conditions were presented was higher than perceived intensity when the eyes- [*M* = 5.31, *SE* = 0.23, 95% CI = (4.83, 5.79)] or mouth-only [*M* = 5.42, *SE* = 0.23, 95% CI = (4.95, 5.89)] conditions were presented, *p*s < 0.018, and no differences were observed between the full-face and eyebrows conditions or between the eyes and mouth conditions (*p*s > 0.446). Refer Supplementary Part 3 for detailed results of perceived intensity of real faces.

The results of the 2 × 4 (type × feature) repeated measures ANOVAs for different expressions showed significant main effects of type, feature, and interaction between them (*F*s > 5.79, *p*s < 0.007, η_p_^2^s > 0.16; Supplementary Figure 2, bottom) in all the expressions. Simple effects tests of happiness revealed that the perceived intensity for the cartoon faces was lower than that for the real faces when the eyes-only (mean difference = −0.88, *SE* = 0.18), mouth-only (mean difference = −0.46, *SE* = 0.19), and full-face (mean difference = −0.48, SE = 0.14) conditions were presented, *p*s < 0.019. For sadness, the perceived intensity for the cartoon faces was higher than that for the real faces for all single features (mean difference > 0.31, *p*s < 0.024). No other differences were observed for happiness and sadness (*p*s > 0.057).

### Discussion

Experiment 2 was conducted to examine the contribution and sufficiency of single facial features for the recognition of emotional expressions in cartoon faces. The sufficiency criterion was examined by presenting only the mouths, eyes, and eyebrows in cartoon faces. We found that the presentation of the mouth alone was sufficient for the identification of happy expressions and that the same level of emotional intensity was perceived when only the mouth was presented as when the full face was presented, which is in line with previous studies on real faces that found the mouth with corners curved upward, characterized by the distinct smiling shape, was a sufficient feature for the recognition of a happy expression (Calvo and Marrero, 2009; Beaudry et al., 2014; Guarnera et al., 2018). A stylized cartoon mouth is also a distinct and key feature for the recognition of happiness. Bimler et al. (2013) found that mouth curvature could express happiness dominantly and could be processed at an early stage and showed signs of implicit categorization. Notably, the superiority of the mouth for conveying happiness was also confirmed by ERP studies (Schyns et al., 2009), and evidence suggests that this may be an effect of low-level feature processing rather than affective processing. For example, Neath-Tavares and Itier (2016) found that when faces with open mouths were presented, better discrimination of a happy expression was displayed, with an early happiness effect starting at P1 but a maximal effect after the peak (115–120 ms); this means that this early effect seems unlikely to reflect a general emotion effect and may be due to the rapid discrimination of a smiling mouth.

Studies that investigated the sufficiency criterion of a single facial feature also revealed the special contribution of eye regions to the recognition of sadness (Nusseck et al., 2008; Calvo and Marrero, 2009; Beaudry et al., 2014; Guarnera et al., 2018). Regarding perceived emotional intensity in cartoon faces, we found that sad eyebrows provided sufficient emotional information to convey the same level of emotional intensity as the full faces. These results reveal the general sufficiency of the eyebrows for cartoon facial expressions. These results are in line with studies that revealed the significance of eyebrows (Lundqvist and Öhman, 2005; Chen et al., 2015). The movement of eyebrows, defined as FACS action units AU1 (inner brow raiser with frontalis, pars medialis), AU2 (outer brow raiser with frontalis, pars lateralis), and AU4 (brow lowerer with corrugator supercilii, depressor supercilii), has been associated with the processing of basic emotional expressions (Cohn et al., 2007). The physical movements of the inner brow raising or outer brow lowering are related to sadness recognition and are conspicuous and readily discerned; thus, they can provide crucial information for emotional expression processing and are sufficient to convey the same level of emotional intensity as the full face.

Additionally, when only single facial features (mouth, eyes, or eyebrows) were presented, (1) sad expressions in cartoon faces were identified more accurately and perceived as sadder than sad expressions in real faces; (2) happy expressions in cartoon faces were identified less accurately for the eyes-only condition and more accurately for the eyebrows-only condition than that in real faces; (3) happy expressions in cartoon faces were perceived as less happy than that in real faces when full face, eyes-, and mouth-only conditions were presented. The results were consistent with previous studies using highly simplified non-real faces, such as schematic and smiley faces, which found that non-real faces could be more effective at conveying negative emotional information, such as threats (Lundqvist and Öhman, 2005; Calvo et al., 2006). However, it should also be noted that happy expressions were identified more accurately in cartoon faces than in real faces when only the eyebrows were presented, which was the opposite of the results of Experiment 1. What is more, the accuracy of the identification of happy expressions in real faces when only the eyebrows were presented was 0.202 and did not differ significantly from chance level (*p* > 0.05), which means that the participants failed to identify the target expression as happiness based only on the information from the eyebrows; thus, the results were inconsistent.

## Experiment 3: Necessity of Specific Facial Features

Experiment 3 was designed to investigate the necessity of specific facial features for the recognition of emotional expressions in cartoon faces. If one feature played a critical role in the recognition of a corresponding emotional expression, the removal of that feature would impair the process of emotion recognition. Thus, a single feature would be considered necessary if a significant difference was found between when it was hidden and when the full face was presented. Previous studies have revealed that the mouth was necessary for happiness recognition and that the eye regions were necessary for sadness recognition (Calder and Jansen, 2005; Calvo and Nummenmaa, 2008; Nusseck et al., 2008; Beaudry et al., 2014). Therefore, in Experiment 3, we hypothesized that the necessity criterion could also be applied to cartoon faces; that is, the mouth is necessary for happiness, and the eyes and eyebrows are necessary for sadness.

### Participants

The estimated sample size for Experiment 3 was 21, with a statistical power of 0.81. We recruited 34 Chinese students from Tsinghua University (female/male: 19/15; mean age ± SD = 21.68 ± 2.96 years old) to take part in the experiment. The exclusion and inclusion criteria were the same as those for Experiment 1.

### Stimuli

In Experiment 3, we concealed facial features (eyebrows vs. eyes vs. mouth) by Gaussian blur at an intensity of 60 (guaranteeing that concealed positions would not be recognizable) and used a size of 60 × 256 pixels (height × width) to ensure that the target facial parts were completely covered. Except for the Gaussian blur of specific features, all stimuli parameters (color, size, brightness, contrast, and resolution) and apparatus were identical to those used in Experiment 1. Finally, 480 images (four concealed cartoons and real features for three emotional expressions in 20 characters) were included in Experiment 3.

### Design and Procedure

Experiment 3 used a 2 (type: cartoon vs. real faces) × 3 (expression: happy vs. neutral vs. sad) × 4 (face without feature: mouth vs. eyes vs. eyebrows vs. full face) within-participants design. The procedure and tasks were the same as those used in Experiment 2.

### Results

Following the same analytical procedure used in Experiment 2, we conducted 2 (type) × 3 (expression) × 4 (face without the feature) repeated measures ANOVAs for expression recognition accuracy and intensity and then decomposed the three-way interaction to further analyze the necessity of facial features for cartoon expression perception.

#### Accuracy of Emotional Facial Expression Recognition

**FIGURE 5:**
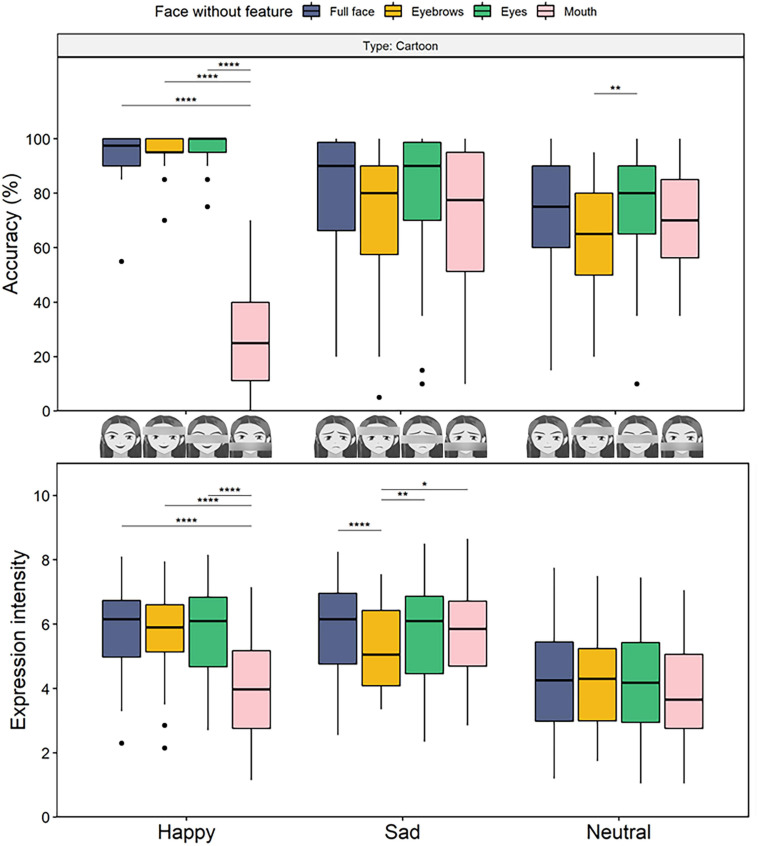
Boxplots for recognition accuracy (top) and perceived expression intensity (bottom) for the assessment of the expressions in cartoon faces as a function of faces without features in Experiment 3. The presentation of faces without mouths damaged the accuracy and intensity rating for happiness, and the presentation of faces without eyebrows decreased the perception of sadness intensity. Here and henceforth stars indicate the following levels of significance: *p < 0.05, **p < 0.01, ***p < 0.001, and ****p < 0.0001, in multiple contrasts, after Bonferroni corrections.

The accuracy results revealed main effects of faces without features [*F*_(2__.45__, 80__.67__)_ = 42.34, *p* < 0.001, η_p_^2^ = 0.56] and expression [*F*_(2, 66)_ = 37.71, *p* < 0.001, η_p_^2^ = 0.53] as well as all two-way and three-way interaction effects (*F*s > 32.39, *p*s < 0.001, η_p_^2^s > 0.49). To specify the necessity of facial features for the accuracy of emotion recognition in cartoon faces (Figure 5, top), we performed a 3 (expression) × 4 (face without the feature) repeated measures ANOVA for cartoon faces and found a main effect of feature concealing and an interaction effect (*F*s < 73.77, *p*s < 0.001, η_p_^2^s > 0.69). Pairwise comparisons revealed that the accuracy of happiness identification was lowest for faces without mouths [*M* = 0.28, *SE* = 0.03, 95% CI = (0.21, 0.35)], *p*s < 0.001, while no difference was observed among the other conditions (*M*s > 0.95, *p*s = 1). For sad expressions, no differences between faces without features and full faces were observed (*p*s > 0.065). Refer to Supplementary Part 3 for detailed results of the accuracy of real faces.

To further compare the necessity of features in cartoon faces with that of real faces, three separate 2 (type) × 3 (face without the feature) repeated measures ANOVAs were performed for the accuracy of different expressions. For happiness, two main effects along with an interaction effect were significant (*F*s > 216.47, *p*s < 0.001, η_p_^2^s > 0.86; Supplementary Figure 4, top); pairwise comparisons showed that the accuracy of recognizing the emotion of happiness in faces without mouths was lower for the cartoon faces [*M* = 0.28, *SE* = 0.03, 95% CI = (0.21, 0.35)] than for the real faces [*M* = 0.94, *SE* = 0.01, 95% CI = (0.91, 0.97), *p* < 0.001], while no significant differences between the cartoon and real faces were observed for the other conditions (*p*s > 0.084). For sadness, two main effects and their interaction effect reached significance (*F*s > 7.64, *p*s < 0.001, η_p_^2^s > 0.18); higher accuracy was observed for the cartoon faces (*M*s > 0.7) than for the real faces (*M*s < 0.5) in all the conditions (*p*s < 0.001), with the differences decreasing from faces without eyes (mean difference = 0.39, *SE* = 0.04) to full faces (mean difference = 0.29, *SE* = 0.04), faces without eyebrows, and faces without mouths (mean difference = 0.22, *SE* = 0.05).

#### Perceived Intensity of Emotional Facial Expressions

The results for intensity (Figure 5, bottom) revealed significant main effects of faces without features [*F*_(3, 99)_ = 13.77, *p* < 0.001, η_p_^2^ = 0.29] and expression [*F*_(2, 66)_ = 64.26, *p* < 0.001, η_p_^2^ = 0.66] and all interaction effects (*F*s > 4.6, *p*s < 0.004, η_p_^2^s > 0.12).

A 3 (expression) × 4 (faces without features) repeated measures ANOVA for cartoon faces revealed two main effects and an interaction effect (*F*s > 13.31, *p*s < 0.001, η_p_^2^s > 0.28). Furthermore, pairwise comparisons showed that the perceived happiness intensity of faces without mouths [*M* = 4.1, *SE* = 0.28, 95% CI = (3.53, 4.66)] was lower than that for full faces and faces without other features (*M*s > 5.72, *p*s < 0.001). Moreover, perceived sadness intensity of faces without eyebrows [*M* = 5.2, *SE* = 0.22, 95% CI = (4.76, 5.65)] was lower than that of other face conditions (*M*s > 5.73, *p*s < 0.015). No other differences were observed in the perceived intensity of the happy and sad expressions (*p*s > 0.313). Refer to Supplementary Part 3 for detailed results of the perceived intensity of real faces.

The three-factor interaction was decomposed by conducting a 2 (type) × 3 (face without the feature) repeated-measures ANOVAs on the expressions. For happy expressions, two main effects of type and interaction were significant (*F*s > 10.19, *p*s < 0.001, η_p_^2^s > 0.23; Supplementary Figure 4, bottom); i.e., the intensity of the cartoon faces was significantly lower than that of the real faces for all the conditions (*p*s < 0.001), and the highest difference was found for faces without mouths (mean difference = −1.87, *SE* = 0.26). For sadness, the effect of type reached significance, with higher perceived intensity reported for the cartoon faces [*M* = 5.65, *SE* = 0.22, 95% CI = (5.2, 6.1)] than for the real faces [*M* = 5.2, *SE* = 0.22, 95% CI = (4.76, 5.65)] in all the conditions, *F*_(1, 33)_ = 29.19, *p* < 0.001, η_p_^2^ = 0.47, and the effect of faces without features [*F*_(3, 99)_ = 20.22, *p* < 0.001, η_p_^2^ = 0.38] indicated a lower intensity for faces without eyebrows [*M* = 4.81, *SE* = 0.21, 95% CI = (4.39, 5.23)] than for the other conditions (*M*s > 5.44), *p*s < 0.001. The interaction between type and faces without features did not reach significance, *F*_(3, 99)_ = 1.69, *p* = 0.173, η_p_^2^ = 0.05.

### Discussion

In Experiment 3, the necessity criterion was examined by hiding the mouth, eyes, and eyebrows of the images, and we found that the mouth was a necessary feature for the recognition of happiness. When the mouth of an image was hidden, the accuracy of expression recognition was significantly impaired for the cartoon faces and perceived emotional intensity was decreased. This is in line with previous studies revealing the necessity of the mouth for the recognition of happy expressions (Nusseck et al., 2008; Calvo and Marrero, 2009; Beaudry et al., 2014; Guarnera et al., 2018).

Another main finding of Experiment 3 was that eyebrows were the only feature that could be considered necessary for the perceived emotional intensity of sad expressions. Many previous studies have focused on the eye region, which includes both the eyes and the eyebrows. When the combined region of the eyes and eyebrows was considered, significant results were found for its necessity for the recognition of sadness in real faces (Nusseck et al., 2008; Calvo and Marrero, 2009; Beaudry et al., 2014; Guarnera et al., 2018). However, in this study, distinct effects were observed for the eyes and eyebrows: whereas the perceived emotional intensity was significantly decreased when the eyebrows were hidden for the identification of sadness, and the cartoon style transform on the eyes did not lead to significant influences. The results of Experiment 3 showed that perceived emotional intensity was significantly decreased when the eyebrows were hidden, which revealed that they were necessary for the perceived emotional intensity of sad expressions in cartoon faces. This was in line with previous studies on schematic faces, which showed that eyebrows, not eyes, were important for conveying general negative emotional information, such as threats (Lundqvist and Öhman, 2005). Previous studies have found that the raising and drawing together of the inner parts of the eyebrows was associated with the recognition of sadness (Ekman and Friesen, 1975; Kohler et al., 2004). Combined with previous fitting models for facial features and expression intensity that highlight the continuous effect of manipulations of the eyebrows on the perceived intensity of sad expressions (Hasegawa and Unuma, 2010), eyebrows may be one of the primary and necessary features for the emotional perception and processing of sad facial expressions.

We also found that sad expressions were recognized more accurately and perceived as sadder in cartoon faces than in real ones. These results are consistent with the findings from Experiments 1 and 2, which revealed the superiority of sad expression recognition in cartoon faces compared with real ones, and these results did not change based on whether the single facial feature was presented or hidden.

## General Discussion

In this study, we investigated the recognition of facial emotional expressions in cartoon faces. The results revealed the presence of a happiness advantage in cartoon faces. Moreover, the sufficiency and necessity of a single facial feature for the recognition of emotional expressions were clarified. The results of the three experiments also revealed a clear difference in perceived intensity across emotions, with sad expressions being perceived as sadder in cartoon faces. However, the results could not lead to a conclusion regarding whether the processing of facial expressions is more feature-based or configuration-based.

### Happiness Recognition Advantage Over Other Emotions

The results of this study revealed a happy expression advantage in cartoon faces, as happiness was identified more accurately than neutral and sad expressions. This is in line with previous studies showing that happiness has superiority over other emotional expressions in real faces (Kirita and Endo, 1995; Leppänen and Hietanen, 2004; Nummenmaa and Calvo, 2015). The results are also consistent with previous studies that used schematic faces and showed that low-level physical differences, characterized by the simplification of real faces to cartoon faces, would not influence this recognition advantage, with happiness being identified faster than disgust or sadness (Leppänen and Hietanen, 2004). However, it should be noted that the emotional expressions that were considered in this study only comprised limited emotion categories, i.e., happiness, sadness, and neutral expressions. Further research is needed to examine whether this effect accounts for different levels of simplification of non-real faces or whether complex asymmetry of emotion recognition exists between real and non-real faces.

### Sadness Perception Superiority of Cartoon Faces

In this study, we found that sad expressions in cartoon faces tended to be perceived as sadder than sad expressions in real faces. In Experiment 1, we proposed the feature processing hypothesis as a possible explanation. The exaggeration and simplification of some facial features in cartoon faces could account for this emotional perception characteristic. When we examined the intensity perception of emotional expressions when a single facial feature was presented or hidden, the results were consistent, and emotion superiority was still shown in Experiments 2 and 3, which suggested that the superior perception of sadness in cartoon faces may be a robust phenomenon independent of specific facial features.

Another explanation presented in Experiment 1 is that the intensity of cartoon sadness expressions might account for the stimulus type itself, as the participants rated the cartoon faces as sadder, regardless of whether they were shown the whole face or some of the facial features. It seems that cartoon faces are more effective in conveying sad information. As indicated by previous studies, the emotional expressions of cartoon and real faces might contain different featural and/or configural information. Mäkäräinen et al. (2014) compared faces with different levels of realism, and they exaggerated the facial expressions with a variety of exaggeration degrees in each level. They found that less realistic faces required more exaggeration to reach the emotional intensity of real faces. Thus, it is possible that the relationship between perceived emotional intensity and physical changes in facial features, which is affected by featural and/or configural information, may be non-linear, e.g., the same degree of lip or eyebrows raising in the cartoon faces may convey a different degree of emotional perception. However, further studies are needed to examine this explanation.

### Sufficiency and Necessity of Single Features

This study provided clear support for the sufficiency and necessity of the mouth for the recognition of happy expressions in cartoon faces. Taken together with the results from Experiments 2 and 3, this shows that information from the mouth itself was sufficient for the participants to identify happy expressions, and the removal of the mouth significantly decreased the accuracy of happiness identification in cartoon faces. This finding is in line with previous studies that investigated the sufficiency and necessity criterion, showing that the mouth should be considered a distinct and salient feature for the recognition of happiness (Nusseck et al., 2008; Calvo and Marrero, 2009; Beaudry et al., 2014; Guarnera et al., 2018). The significance of the mouth is diminished when this feature is artistically stylized, as in cartoon faces. Moreover, to extend the previous findings, the results show that the participants could perceive the same level of emotional intensity when viewing the mouth alone as they could when viewing the full face and hiding the mouth decreased the perceived emotional intensity of happiness. The unique role of the mouth applied not only to recognition accuracy, identification time, and fixation time, as found in previous studies (Nusseck et al., 2008; Calvo and Marrero, 2009; Beaudry et al., 2014; Guarnera et al., 2018), but also to the emotional information it conveys, suggesting that the perception of happiness could be based specifically on the shape of the mouth when smiling and the muscle movement around it.

Another main finding in this study was that in examining the sufficiency and necessity criteria of eyes and eyebrows separately, we showed that only the eyebrows had an important role in the perceived emotional intensity of sad expressions. When only the eyebrows were presented, the perceived intensity of sadness was not different from that when the full face was presented, but the perceived intensity was significantly affected when the eyebrows were hidden. This is in line with previous results showing that eyebrows have increased significance for conveying negative emotional information, such as threats (Lundqvist and Öhman, 2005). Previous fitting models for facial features and expression intensity have also highlighted the continuous effect of manipulations of the eyebrows on the perceived intensity of sad expressions (Hasegawa and Unuma, 2010). The results may particularly be relevant to the shape of eyebrows in the sad cartoon style, and in this study, the inward eyebrow ends were raised in an exaggerated way, which might have an impact on the obtained results. Another critical factor that should be taken into consideration is that the findings of this study might not be generalized to the detection of cartoon expressions in non-Eastern cultures where observers are less likely to judge facial emotions with the upper part of the face (Yuki et al., 2007; Jack et al., 2009; Wingenbach et al., 2020).

## Limitation and Future Directions

Although this study provides an insight into the emotional facial expression recognition of cartoon faces, it has several limitations. First, as a preliminary study, we only take into account three emotional expressions (happy, sad, and neutral), because the sufficiency and necessity criteria of these emotions have been well replicated in previous studies on real faces. However, other basic emotions, such as anger, fear, surprise, and disgust (Ekman and Friesen, 1978), for which the existing research shows inconsistent results (Nusseck et al., 2008; Calvo and Marrero, 2009; Beaudry et al., 2014; Guarnera et al., 2018), were not included in this study. Thus, future investigations are needed to examine the contributions of single features in cartoon faces to these emotion categories. Second, we found that cartoon faces with sad expressions were perceived as sadder and were recognized more readily than real faces with sad expressions, and this could not be explained by the exaggeration of single features. However, it remains unclear what caused this superiority and whether cartoon faces are superior only for the presentation of sadness or for negative emotional information in general. Third, the results of this study were derived entirely from behavioral experiments; therefore, we cannot draw any conclusions regarding the processing mechanism because of the lack of psychophysiological data (e.g., eye tracking, EEG). Further research can incorporate these data to explore the holistic and feature accounts of the recognition of emotional facial expressions in cartoon faces. Fourth, although the cartoon faces used in this study were generated with a computational model (Kim et al., 2019) and an artist manually modulated the cartoon emotional expressions to match their real counterparts, the similar degrees of physical deformation of single features (e.g., the similarity of the curvature of the eyebrows) still could not ensure that the emotional information conveyed by the two types of faces was equivalent. A wealth of evidence has shown that genuineness could play an important role in emotion perception. Fifth, although the cartoon facial features were masked by Gaussian blur following the classical paradigm (Beaudry et al., 2014), the results could be generalized to similar situations in which certain cartoon facial features are covered with clothing such as scarves, masks, or sunglasses, especially during the current pandemic (Kret and De Gelder, 2012; Calbi et al., 2021). Further studies are necessary to explore the validity of the findings in real-world scenarios that contain rich and dynamic social information. Finally, although the results of this study showed the key role of the mouth and eyebrows for the successful recognition of happiness and sadness, respectively, the influence of the interplay among the different facial features on the recognition of various facial expressions cannot be ignored. Therefore, the role of the individual features in the recognition of emotions in both cartoon faces and real (or morphed) images should be further studied in the future (Bimler and Paramei, 2006).

## Conclusion

To investigate facial emotional expression recognition in cartoon faces, three experiments were performed in this study. We found that the processing of emotion in cartoon faces showed a happiness advantage and that the highest recognition accuracy was obtained for happy expressions in cartoon faces. In terms of perceived intensity, cartoon faces with sad expressions were perceived as sadder than real faces with sad expressions. Furthermore, facial features showed a dissimilar impact on the recognition of emotional facial expressions, and we highlighted the role of the mouth in happiness recognition and the role of the eyebrows in sadness recognition. This study provides an important reference for extending existing facial emotion recognition studies, from real faces to cartoon faces, and the importance of features that was revealed in this study may shed light on the development of cartoon characters for emotional and social artificial intelligence.

## Data Availability Statement

The original contributions presented in the study are included in the article/Supplementary Material, further inquiries can be directed to the corresponding author/s.

## Ethics Statement

The studies involving human participants were reviewed and approved by the Institutional Review Board of the Department of Psychology at Tsinghua University. The patients/participants provided their written informed consent to participate in this study. Written informed consent was not obtained from the individual(s) for the publication of any potentially identifiable images or data included in this article.

## Author Contributions

Y-JL and DZ: conceptualization, project administration, and funding acquisition. SZ, XL, DZ, and Y-JL: methodology. SZ, XL, YS, and NL: stimuli preparation and program writing. SZ, XL, and XY: writing and preparation of the original draft, formal analysis, and investigation. SZ, XL, XY, DZ, and Y-JL: data curation, writing, review, and editing. DZ and Y-JL: supervision. All authors contributed to the article and approved the submitted version.

## Conflict of Interest

The authors declare that the research was conducted in the absence of any commercial or financial relationships that could be construed as a potential conflict of interest.

## Publisher’s Note

All claims expressed in this article are solely those of the authors and do not necessarily represent those of their affiliated organizations, or those of the publisher, the editors and the reviewers. Any product that may be evaluated in this article, or claim that may be made by its manufacturer, is not guaranteed or endorsed by the publisher.
