# Supplementary figures and images for: The Influence of Key Facial Features on Recognition of Emotion in Cartoon Faces
Source: Front Psychol. 2021 Aug 10;12:687974. doi: 10.3389/fpsyg.2021.687974 (PMC8382696; doi:10.3389/fpsyg.2021.687974)

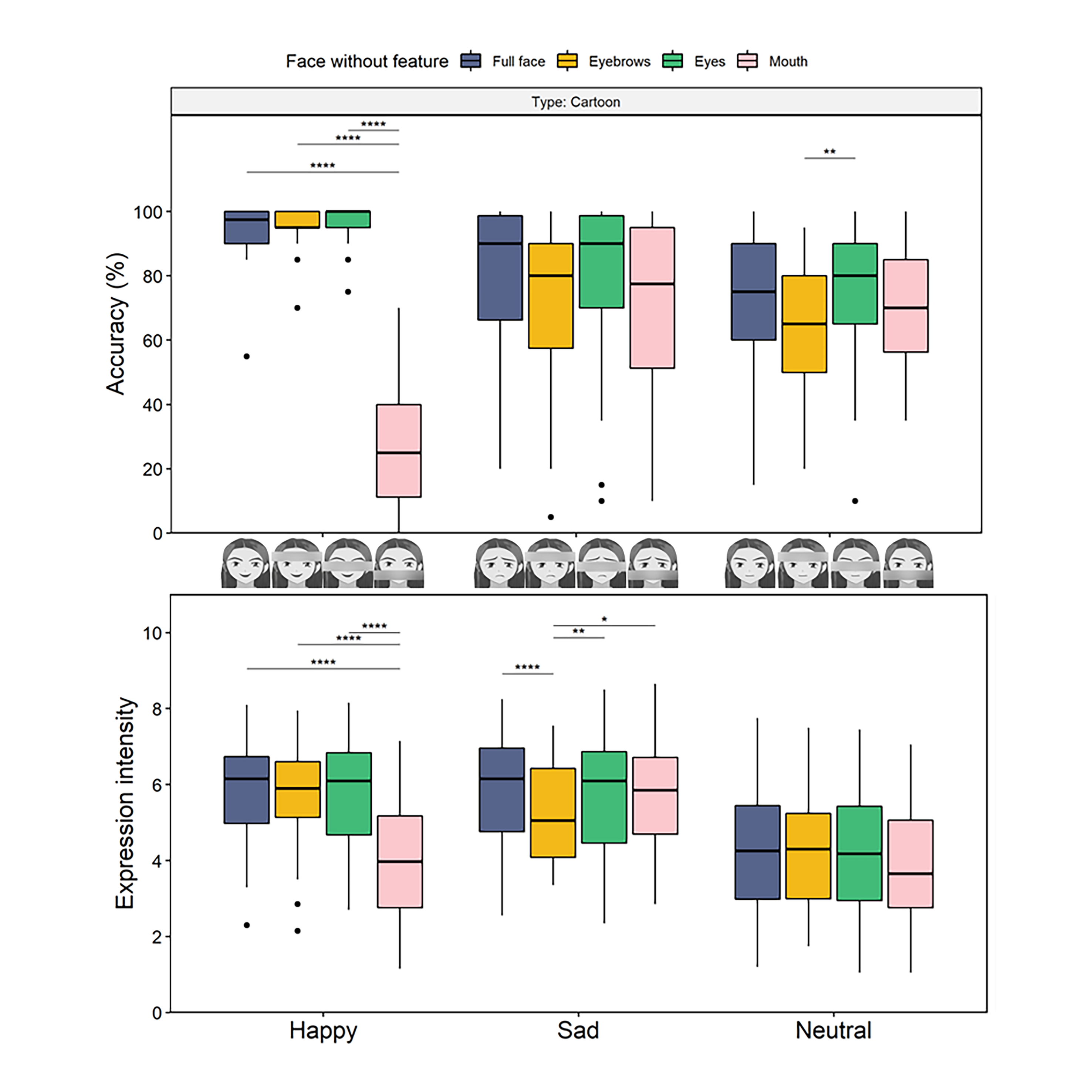

Supplement: Supplementary file 2 [file Data_Sheet_2.ZIP › Figures/Figure 5.tif]

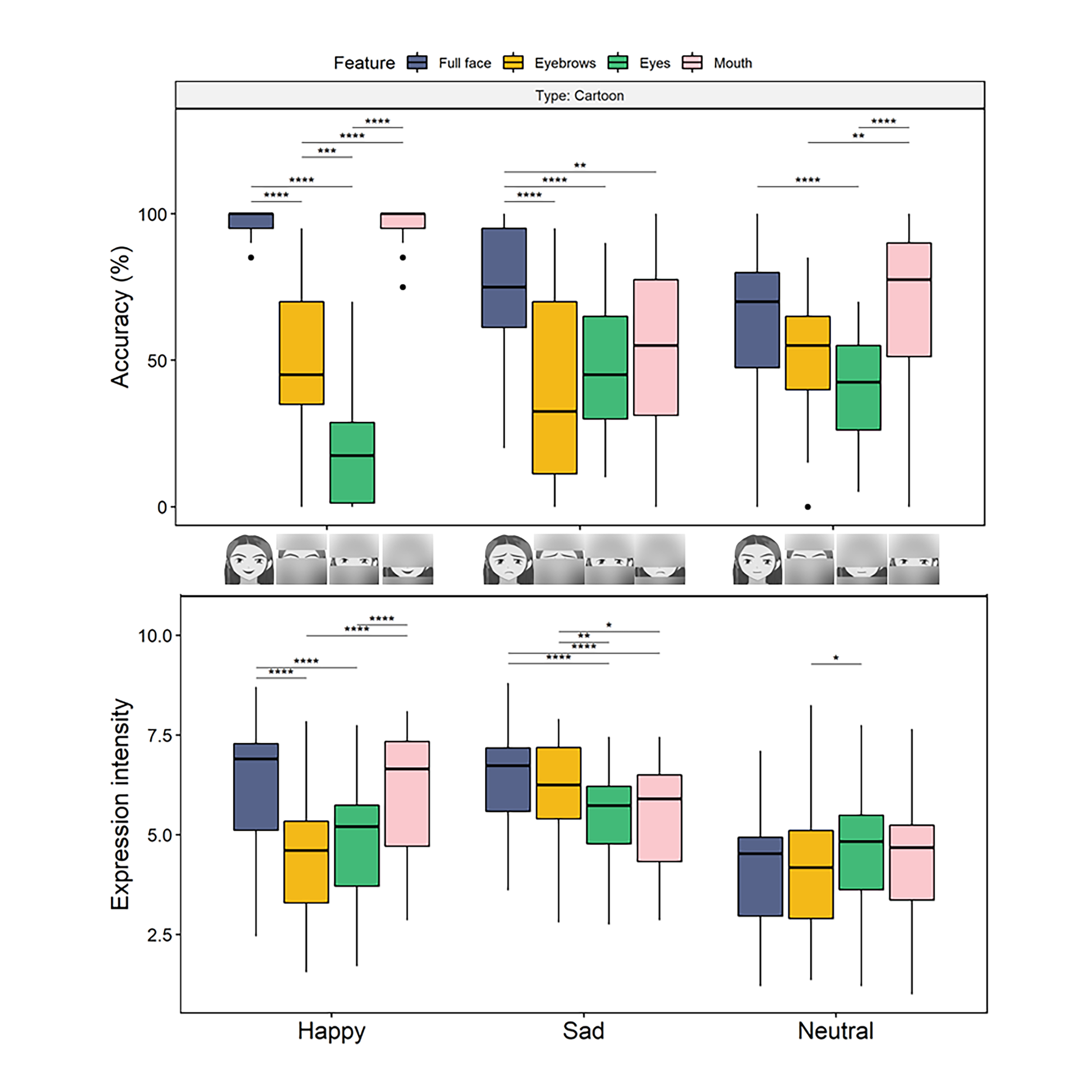

Supplement: Supplementary file 2 [file Data_Sheet_2.ZIP › Figures/Figure 4.tif]

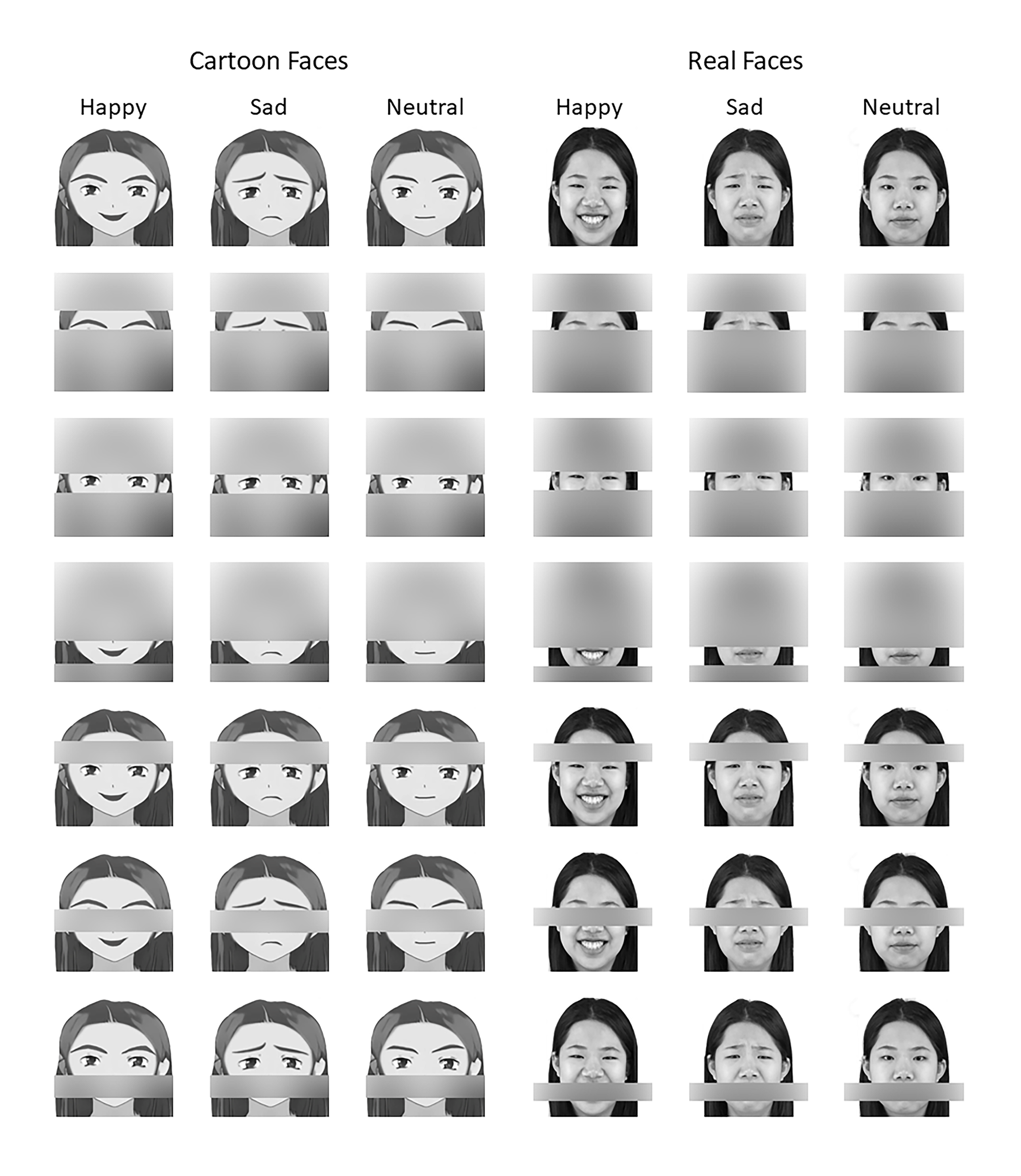

Supplement: Supplementary file 2 [file Data_Sheet_2.ZIP › Figures/Figure 1.tif]

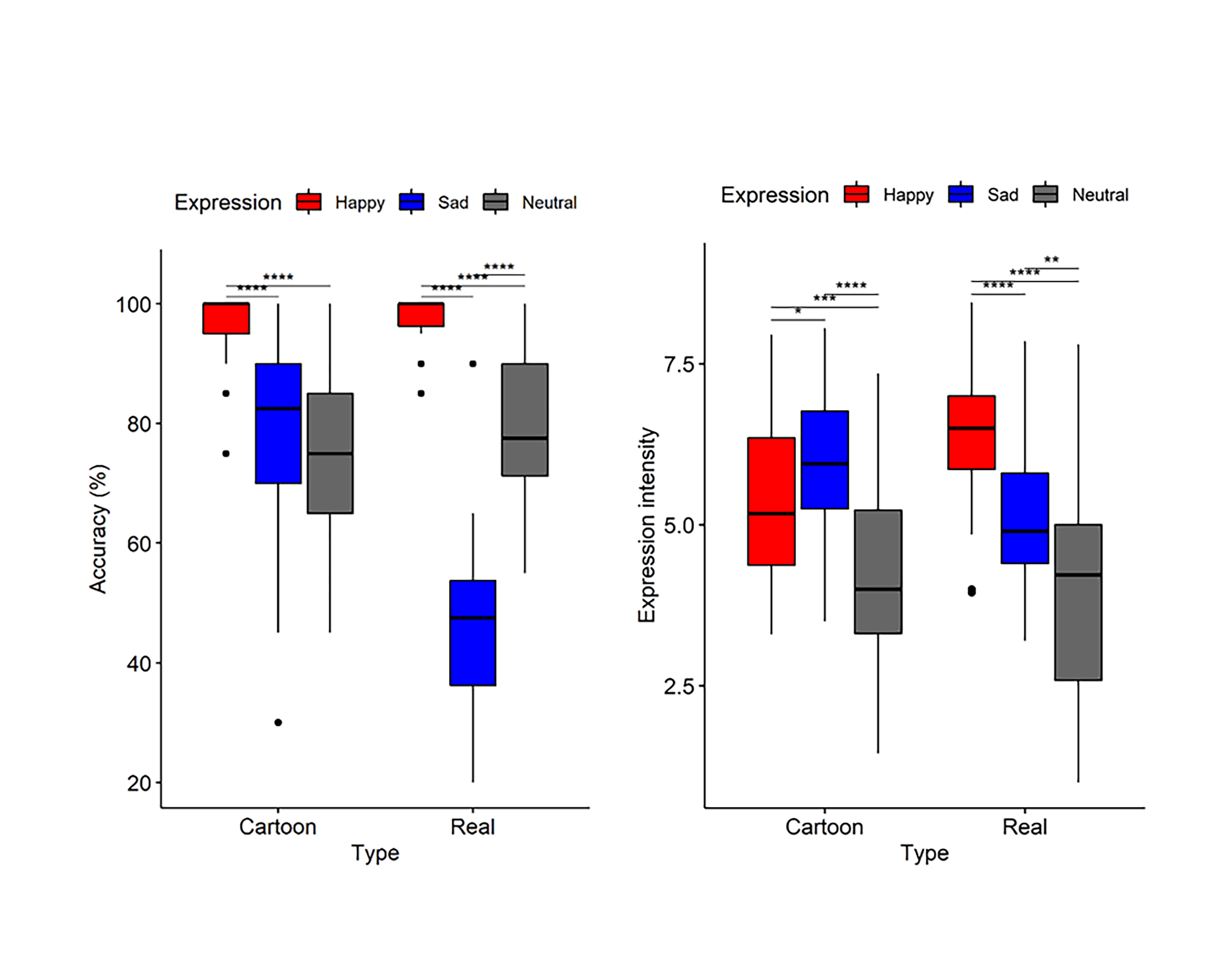

Supplement: Supplementary file 2 [file Data_Sheet_2.ZIP › Figures/Figure 3.tif]

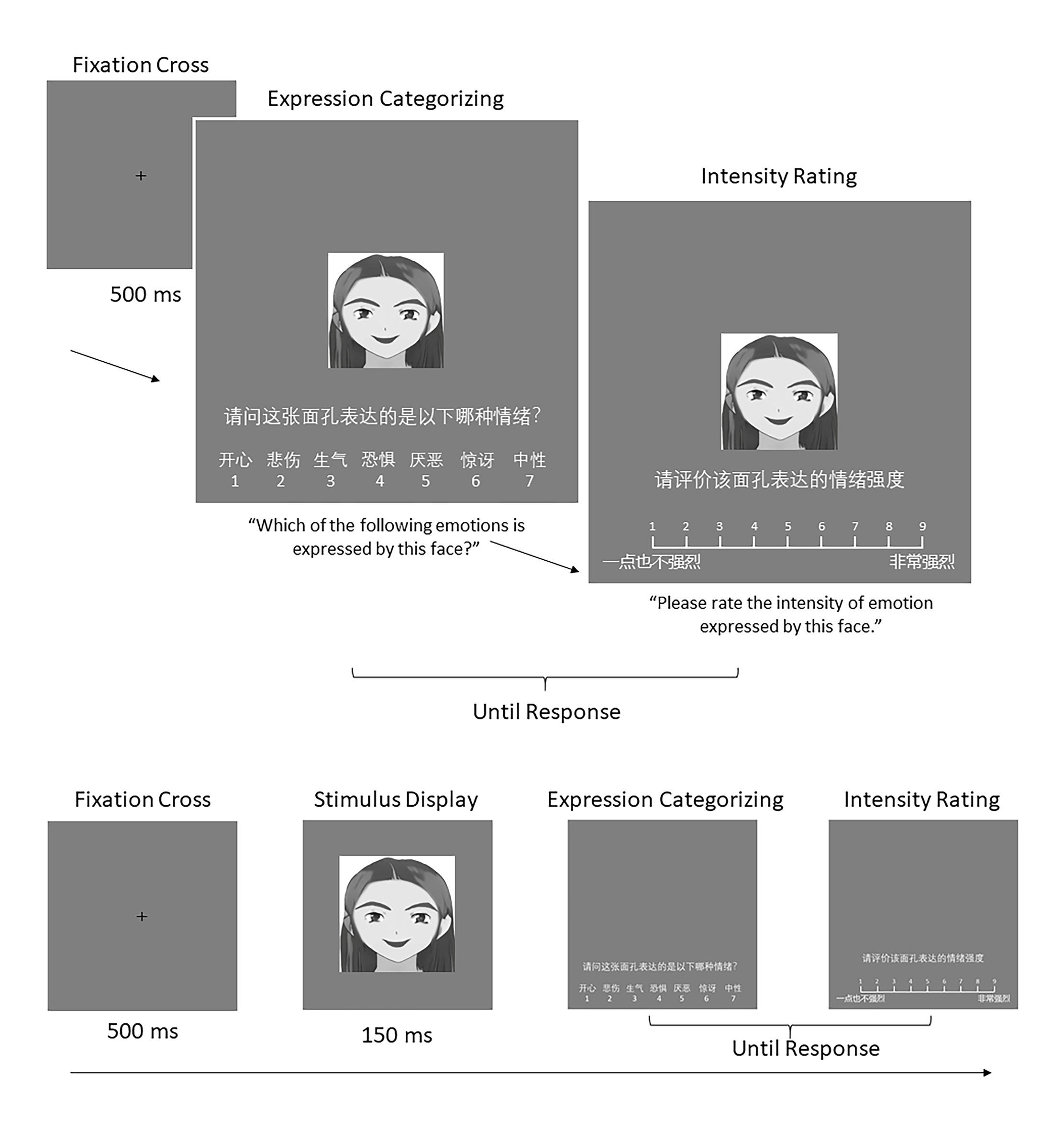

Supplement: Supplementary file 2 [file Data_Sheet_2.ZIP › Figures/Figure 2.tif]

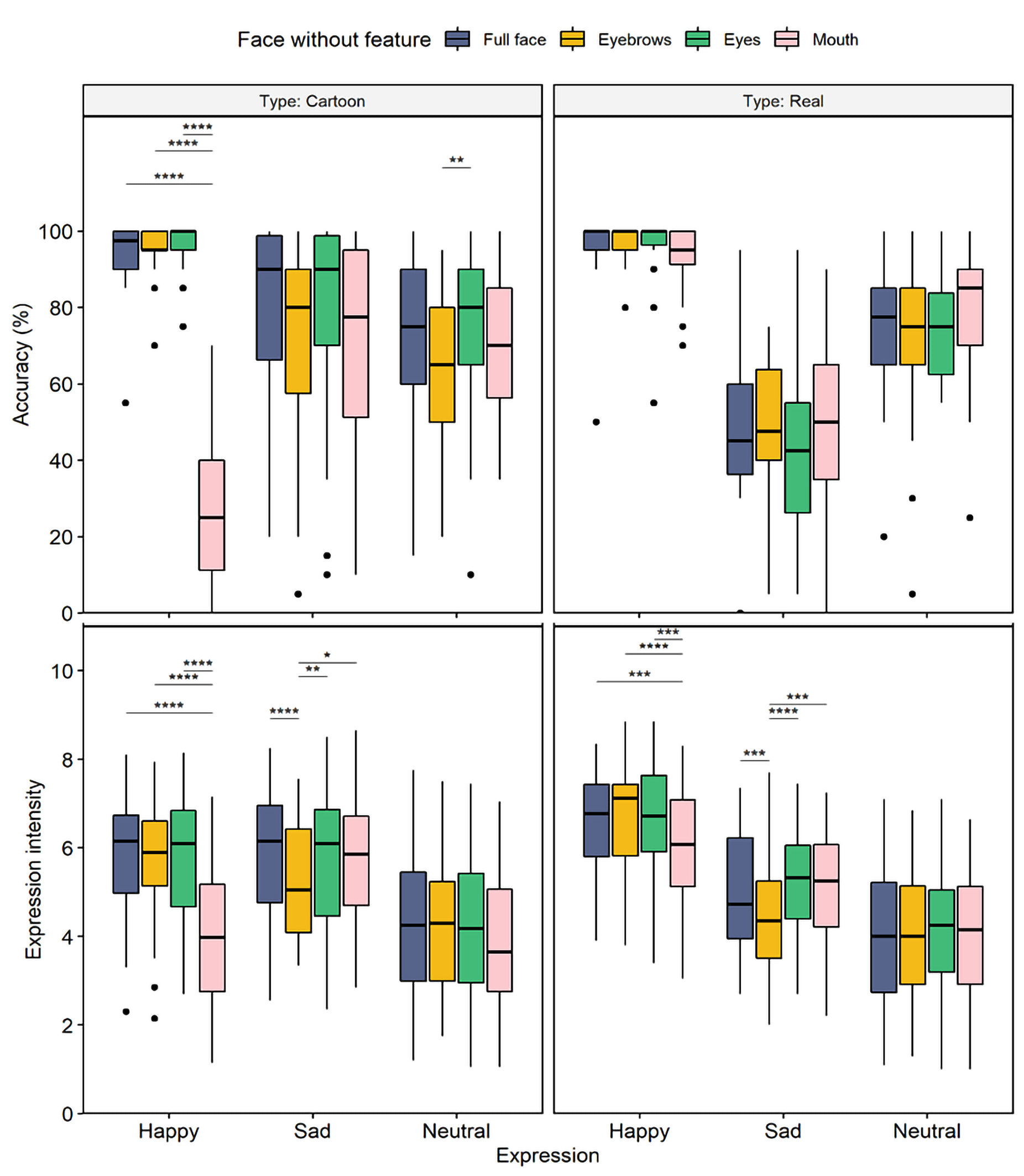

Supplement: Supplementary file 2 [file Data_Sheet_2.ZIP › Figures/Figure s4.tif]

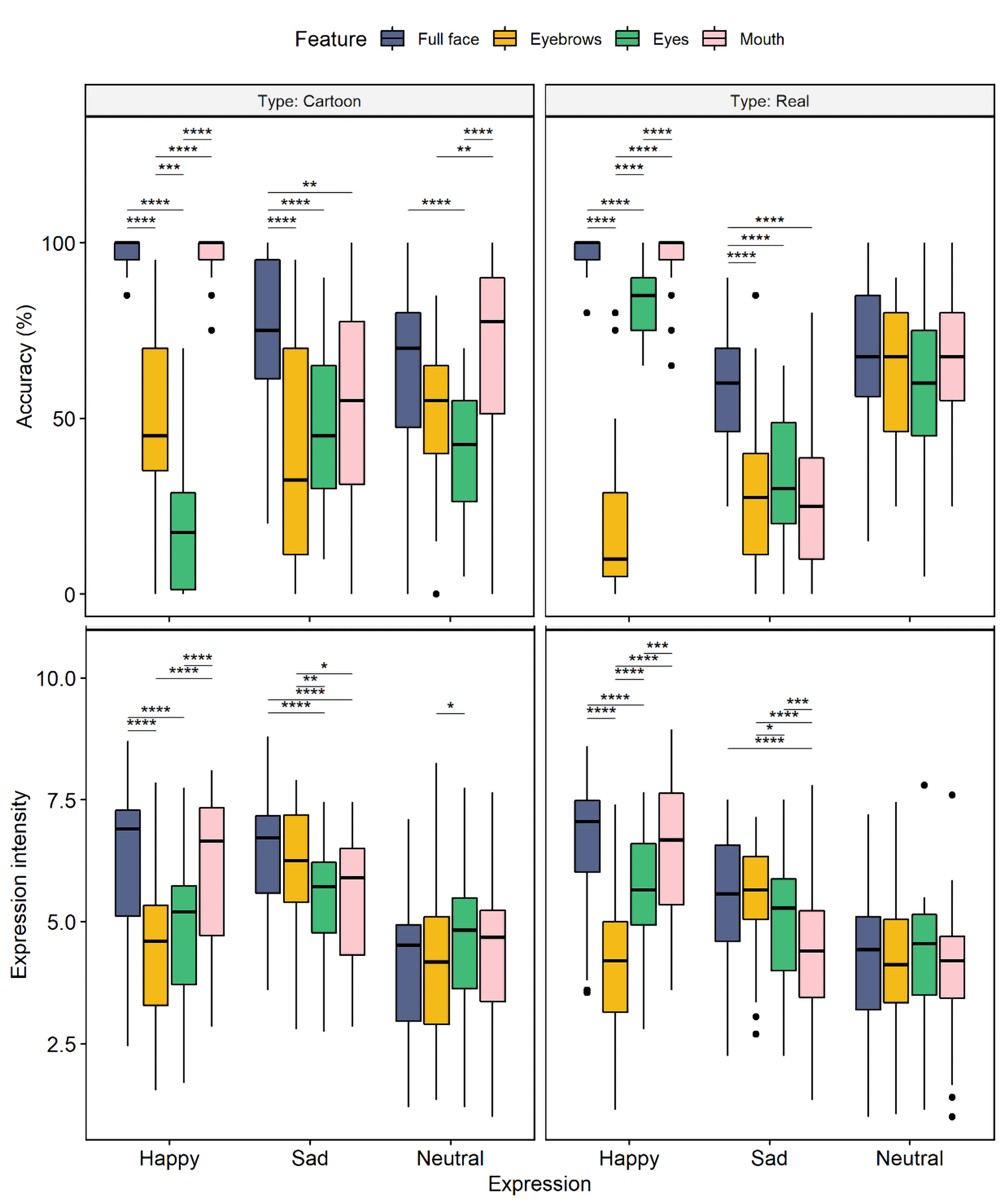

Supplement: Supplementary file 2 [file Data_Sheet_2.ZIP › Figures/Figure s2.tif]

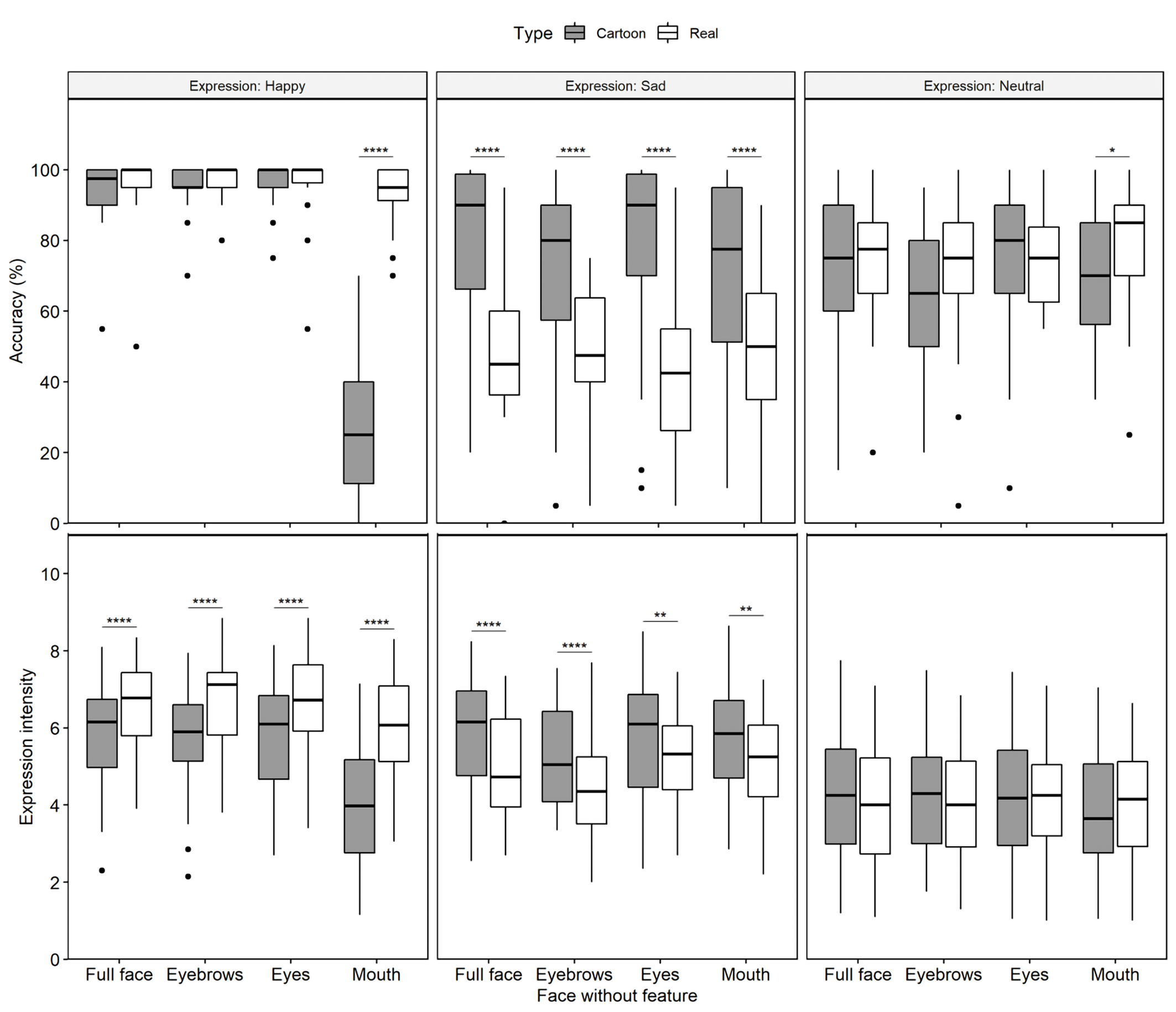

Supplement: Supplementary file 2 [file Data_Sheet_2.ZIP › Figures/Figure s3.tif]

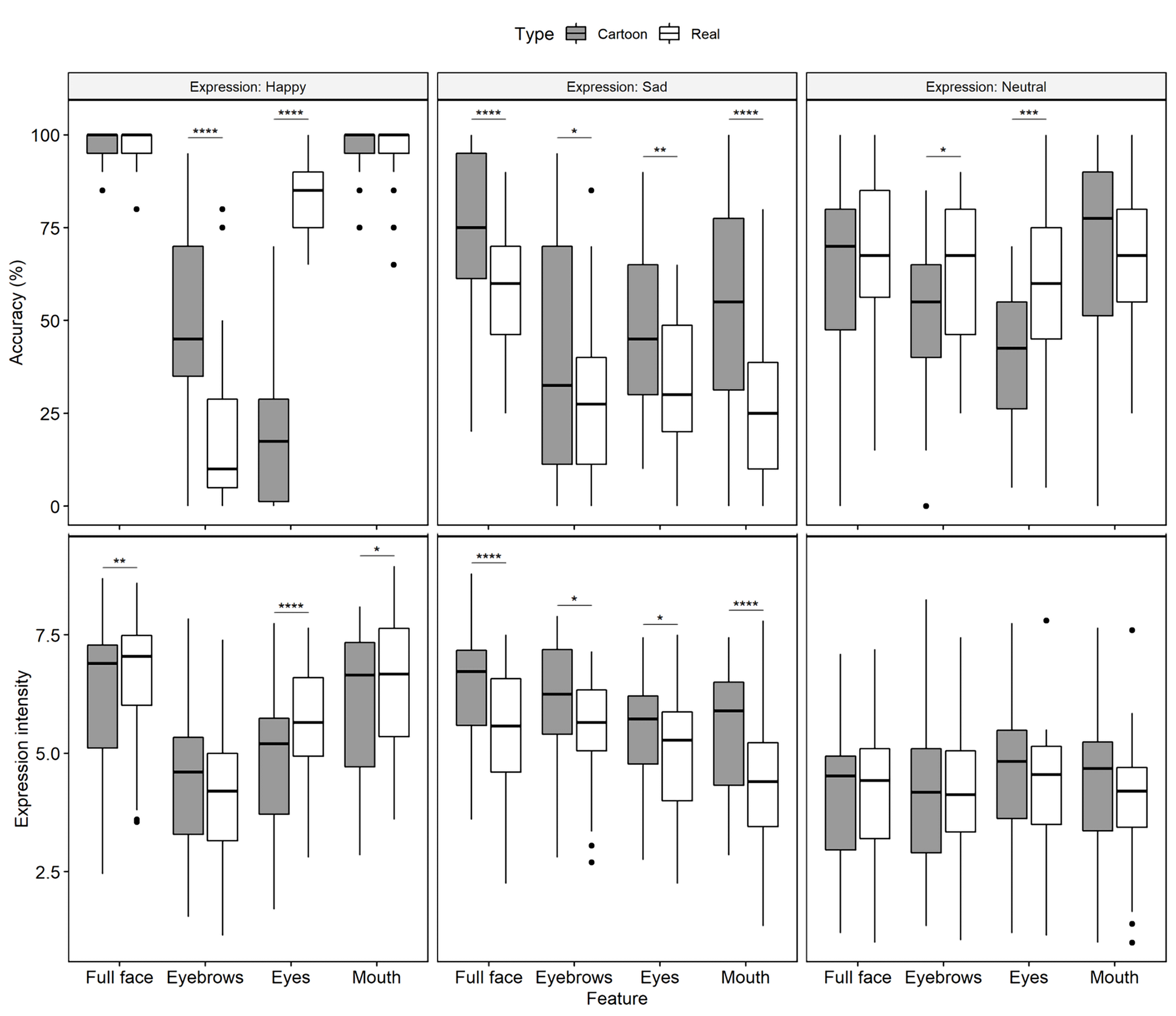

Supplement: Supplementary file 2 [file Data_Sheet_2.ZIP › Figures/Figure s1.tif]
