# Supplementary material for: The Influence of Key Facial Features on Recognition of Emotion in Cartoon Faces
Source: Front Psychol. 2021 Aug 10;12:687974. doi: 10.3389/fpsyg.2021.687974 (PMC8382696; doi:10.3389/fpsyg.2021.687974)
Supplement: Supplementary file 3 [file Table_1.DOCX]

Supplementary Material

# Participants, Procedure and Results in Pre-experiment

Thirty Chinese participants (female/male: 17/13; mean age ± *SD* = 22.20 ± 2.64 years old) were recruited for pre-experiment.

One hundred and twenty facial images (20 exemplars × 2 types × 3 expressions) were resized to a size of 300× 300 pixels with brightness adjusted to the average of their initial ones. Each trial began with a a central fixation for 500 ms, then a facial image appeared and remained until the participant responded. Below the facial image, the words “positive, neutral, negative” were presented on the bottom center of the screen. These words were used instead of “happy, sad and neutral” for collecting the participants’ responses on the general valence levels of the cartoon faces, as the transition from real faces to cartoon faces could have induced possible changes in the specific emotional categories. And a further investigation on this issue was conducted in the formal experiments, where more emotion categories were used as the response options. Participants were required to categorize the emotion of the facial image by clicking the corresponding location of the word. Cartoon and real faces were presented in different blocks in a counterbalanced order across participants. Images performed by different exemplars and expressions were presented randomly within each block. The data of pre-experiment was collected on website, in which the front-end was programmed by using JavaScript and back-end was programmed by using Python (<https://github.com/Nicki-Liu/Emotion/tree/main/Webserver>). Participants had earned appropriated reward after completing the experiment.

All contrasts were Bonferroni corrected for multiple comparisons, and it was considered statistically significant when *p*-value < .05. Greenhouse-Geisser correction was applied when the sphericity hypothesis was violated. The results of a 2 (type) × 3 (expression) repeated-measure ANOVA on accuracy revealed significant main effects of expression (*F*_(1.16, 33.65)_ = 4.64, *p* = .033, η_p_^2^ = .14) and interaction between expression and type (*F*_(1.45, 41.90)_ = 9.05, *p* < .002, η_p_^2^ = .24); the main effect of type did not reach significance, *F*_(1, 29)_ = 0.70, *p* = .410, η_p_^2^ = .02. Pairwise comparisons between expressions showed the accuracy of happiness (*M* = 0.98, *SE* = 0.01, 95%CI = [0.97, 1.00]) and sadness (*M* = 0.95, *SE* = 0.01, 95%CI = [0.93, 0.97]) were higher than that of neutral expression (*M* = 0.92, *SE* = 0.03, 95%CI = [0.87, 97]), *p*s < .063, while no difference was found between happiness and sadness, *p* = .883. Results of the simple effect analysis for interaction effect showed that for cartoon faces, the accuracy of happiness (*M* = 0.98, *SE* = 0.01, 95%CI = [0.97, 0.99]) and sadness (*M* = 0.98, *SE* = 0.01, 95%CI = [0.96, 1.00]) were higher than that of neutral expression (*M* = 0.91, *SE* = 0.03, 95%CI = [0.84, 0.96]), *p*s < .032; for real faces, the accuracy of happiness (*M* = 0.99, *SE* = 0.01, 95%CI = [0.96, 1.00]) was higher than that of sadness (*M* = 0.91, *SE* = 0.02, 95%CI = [0.88, 0.95, *p* < .001]), while there were no differences between happy/sad expression and neutral expression (*M* = 0.94, *SE* = 0.03, 95%CI = [0.89, 1.00], *p*s > .360). Results of another direction for decomposing the interactive effect showed higher accuracy of recognizing sadness for cartoon faces than for real faces (mean difference = 0.07, *SE* = 0.02), *p* < .001. No other differences were observed between types in the accuracy of recognizing happiness and neutral expression, *p*s > .124.

1. **Responses Analyses for Recognition of Emotional Expressions in Experiment 1, 2 and 3**

To analyze the specific responses of recognition of emotional expressions, we calculated the percentages of seven emotional categories (happy, sad, angry, disgust, fear, surprise, and neutral) separately choosen by participants. The main purpose of these analyses is to test whether the percentages of wrong responses would be higher than the chance level (1/7 = 0.14), thus for setting a loose criterion to detect the significance, we did one-sample T testes for each categories with the chance level and not adjust the *p*-value for multiple comparisons.

- 1. *Experiment 1*

The total number of one-sample T tests was 42 (7 responses * 2 types * 3 expressions). Results revealed that all of the percentage of the correct response was significantly higher than chance level (mean difference > 0.32, *p*s < .001); for choosing the six wrong emotional categories, the percentage of digust for recognizing sadness of real faces was higher than the chance level (*M* = 0.28, *SE* = 0.04, 95%CI = [0.21, 0.36], *p* < .001), whereas other wrong response were lower than chance level, *p*s < .001, or not differed from the chance level, *p*s > .470.

- 1. *Experiment 2*

The total number of one-sample T tests was 168 (7 responses * 2 types * 3 expressions * 4 features). The results of the correct responses revealed that the percentages in other conditions were higher than it (mean difference > .11), *p*s < .001, except that the correct response for the “real faces”-happiness-eyebrows condition was not differed from the chance level (*M* = 0.19, *SE* = 0.04, 95%CI = [0.12, 0.27], *p* = .189).

For the six wrong responses of real-faces conditions, the percentages of choosing “disgust” in sadness-eyes condition (*M* = 0.21, *SE* = 0.03, 95%CI = [0.16, 0.27]), choosing “angry” (*M* = 0.19, *SE* = 0.02, 95%CI = [0.15, 0.23]) and “disgust” (*M* = 0.22, *SE* = 0.03, 95%CI = [0.15, 0.29]) in sadness-eyebrows condition, and choosing “neutral” in sadness-mouths condition (*M* = 0.38, *SE* = 0.03, 95%CI = [0.32, 0.44]) were higher than the chance level, mean difference > 0.04, *p*s < .036.

For the wrong responses of cartoon-faces conditions, the percentages of choosing “neutral” in happiness-eyes condition (*M* = 0.26, *SE* = 0.04, 95%CI = [0.18, 0.35]), and choosing “neutral” in happiness (*M* = 0.38, *SE* = 0.03, 95%CI = [0.32, 0.44]), choosing “happy” in neutral expressions (*M* = 0.26, *SE* = 0.04, 95%CI = [0.18, 0.35]) and choosing “disgust” in sadness (*M* = 0.27, *SE* = 0.05, 95%CI = [0.17, 0.37]) of eyebrows condition were higher than chance level, mean difference > 0.13, *p*s < .012.

The percentages of other wrong responses were not differed from the chance level, *p*s > .072, or lower than it, *p*s < .001.

- 1. *Experiment 3*

The total number of one-sample T tests was 168 (7 responses * 2 types * 3 expressions * 4 face without features). The results of the correct responses revealed that all of the percentages were significantly higher than the chance level, mean difference > 0.13, *p*s < .001. For the percentages of choosing the six wrong responses, the reponses of choosing “neutral” in cartoon-happiness-“face without mouths” condition (*M* = 0.58, *SE* = 0.04, 95%CI = [0.50, 0.66]), and that of choosing “disgust” in “real faces”-sadness-“face without eyes” condition (*M* = 0.27, *SE* = 0.03, 95%CI = [0.20, 0.34]) and “real faces”-sadness-“full faces” condition (*M* = 0.20, *SE* = 0.02, 95%CI = [0.16, 0.25]) were higher than chance level, mean difference > 0.06, *p*s < .012. Other percentages of responses were not differed from chance level, *p*s > .108, or lower than it, *p*s < .001.

1. **Results of real faces in Experiments 2 and 3**
   1. *Accuracy and perceived intensity of real faces in Experiment 2*

We conducted 2 (type) × 3 (expression) × 4 (feature) repeated-measure ANOVAs for accuracy and intensity separately. The three-way interaction was decomposed by splitting the type to specify the effect of features in real emotional facial expression. For the results of 2 × 3 × 4 repeated-meature ANOVA and cartoon faces, please see the formal manuscript for details.

The results of a 3 (expression) × 4 (feature) repeated-measure ANOVA for the accuracy of the recognition of real emotional faces revealed the main effects of feature and expression coupled with an interaction effect between them (*F*s > 66.22, *p*s < .001, η_p_^2^s > .65, see the upper right of Supplementary Figure 1). The pairwise comparisons showed that, for happiness recognition, the accuracy did not differ between the mouth-only (*M* = 0.96, *SE* = 0.02, 95%CI = [0.93, 0.99]) and full-face conditions (*M* = 0.97, *SE* = 0.01, 95%CI = [0.96, 0.99], *p* = 1.000), and both yielded greater accuracy than when only the eyebrows (*M* = 0.20, *SE* = 0.03, 95%CI = [0.13, 0.28]) or eyes (*M* = 0.84, *SE* = 0.02, 95%CI = [0.80, 0.88], *p*s < .001) were presented (*p*s < .001). For sadness recognition, the accuracy was higher for the full-face condition (*M* = 0.59, *SE* = 0.03, 95%CI = [0.53, 0.65]) than when any single facial feature was presented alone (*M*s < 0.32, *p*s < .001), and no differences were observed among the single features that were presented separately (*p*s = 1.000). It should be noted that the results of 2 (type) × 3 (expression) × 4 (feature) repeated-measure ANOVA for the accuracy revealed the significant interaction between type and feature, *F*_(3, 87)_ = 62.20, *p* < .001, η_p_^2^ = .68, indicating that the large difference of accuracy between the features-only and full-faces condition was found for cartoon faces (*F*_(3, 27)_ = 178.67, *p* < .001, η_p_^2^ = .95) than that for real faces (*F*_(3, 27)_ = 129.83, *p* < .001, η_p_^2^ = .39).

The results of a 3 (expression) × 4 (feature) repeated-measure ANOVA for the perceived intensity of real emotional faces revealed the significant effects of features, expressions, and a interaction between them (*F*s > 23.09, *p*s < .001, η_p_^2^s > .44, see the lower right of Supplementary Figure 1). The pairwise comparisons showed that, for happiness, the perceived intensity was higher for mouth-only (*M* = 6.47, *SE* = 0.29, 95%CI = [5.88, 7.06]) and full-faces condition (*M* = 6.68, *SE* = 0.27, 95%CI = [6.13, 7.23]) than for eyes-only condition (*M* = 5.58, *SE* = 0.22, 95%CI = [5.13, 6.03]), then to that for eyebrows-only condition (*M* = 3.95, *SE* = 0.28, 95%CI = [3.37, 4.52]), *p*s < .001. For the sadness, the perceived intensity for full-faces (*M* = 5.53, *SE* = 0.25, 95%CI = [5.01, 6.04]) and eyebrows-only condition (*M* = 5.61, *SE* = 0.22, 95%CI = [5.16, 6.07]) were higher than that for eyes-only condition (*M* = 5.01, *SE* = 0.22, 95%CI = [4.57, 5.45]), and both yielded higher intensity rating than when only the mouths were presented (*M* = 4.30, *SE* = 0.25, 95%CI = [3.78, 4.82]), *p*s < .027; no differences were observed in comparing that for full face and eyebrows (*p* = 1.000). No significant difference between feature conditions was observed for perceiving neutral intensity (*p*s > .105). The 2 × 3 × 4 repeated-measure ANOVA for the perceived intensity also revealed the significant interaction between type and feature, *F*_(3, 87)_ = 2.95, *p* = .037, η_p_^2^ = .09, suggesting that the perceived intensity of real faces (*F*_(3, 27)_ = 18.44, *p* < .001, η_p_^2^ = .67) was more affected by features than that of cartoon faces (*F*_(3, 27)_ = 9.76, *p* < .001, η_p_^2^ = .52).

- 1. *Accuracy and perceived intensity of real faces in Experiment 3*

We conducted 2 (type) × 3 (expression) × 4 (face without feature) repeated-measure ANOVAs for accuracy and intensity separately. The three-way interaction was decomposed by splitting the type to specify the effect of features in real emotional facial expression. For the results of 2 × 3 × 4 repeated-meature ANOVA and cartoon faces, please see the formal manuscript for details.

The results of a 3 (expression) × 4 (face without feature) repeated-measure ANOVA for the accuracy of real emotional faces only revealed a significant main effects of expressions was found in real-man faces (*F*_(1.69, 55.77)_ = 166.05, *p* < .001, η_p_^2^ = 0.83). No other main effect or interanction was found, *F*s < 2.98, *p*s > .058, see the upper right of Supplementary Figure 3.

The results of a 3 (expression) × 4 (face without feature) repeated-measure ANOVA for the perceived intensity of real emotional faces revealed the significant effects of face without features, expressions, and a interaction between them (*F*s > 3.68, *p*s < .001, η_p_^2^s > .10, see the lower right of Supplementary Figure 3). The pairwise comparisons showed that, for the happiness, the perceived intensity was lowest for face without mouth (*M* = 5.96, *SE* = 0.23, 95%CI = [5.50, 6.43]) than for full-faces condition (*M* = 6.54, *SE* = 0.21, 95%CI = [6.11, 6.98]) followed for face without eyes (*M* = 6.69, *SE* = 0.22, 95%CI = [6.24, 7.13]), then to that for face without eyebrows condition (*M* = 6.70, *SE* = 0.21, 95%CI = [6.27, 7.13]), *p*s < .001. For the sadness, the perceived intensity for face without eyebrows (*M* = 4.41, *SE* = 0.22, 95%CI = [3.97, 4.87]) were lowest than that for full face (*M* = 5.03, *SE* = 0.21, 95%CI = [4.59, 5.46]), face without eyes (*M* = 5.17, *SE* = 0.22, 95%CI = [4.73, 5.61]) and face without mouth conditions (*M* = 5.16, *SE* = 0.23, 95%CI = [4.70, 5.63]), *p*s < .0001. No differences were observed in comparing that for full face and face without eyes and mouth (*p*s = 1.000). No significant difference between feature conditions was observed for perceiving neutral intensity (*p*s = 1.000). The 2 × 3 × 4 repeated-measure ANOVA for the perceived intensity also revealed the significant interaction between type and feature, *F*_(3, 87)_ = 4.91, *p* = .007, η_p_^2^ = .13, suggesting that the persived intensity of cartoon faces (*F*_(3, 31)_ = 8.79, *p* < .001, η_p_^2^ = .46) was more affected by the mask of features than that of real faces (*F*_(3, 31)_ = 3.22, *p* = .036, η_p_^2^ = .24).

1. **Supplementary Figures**

**
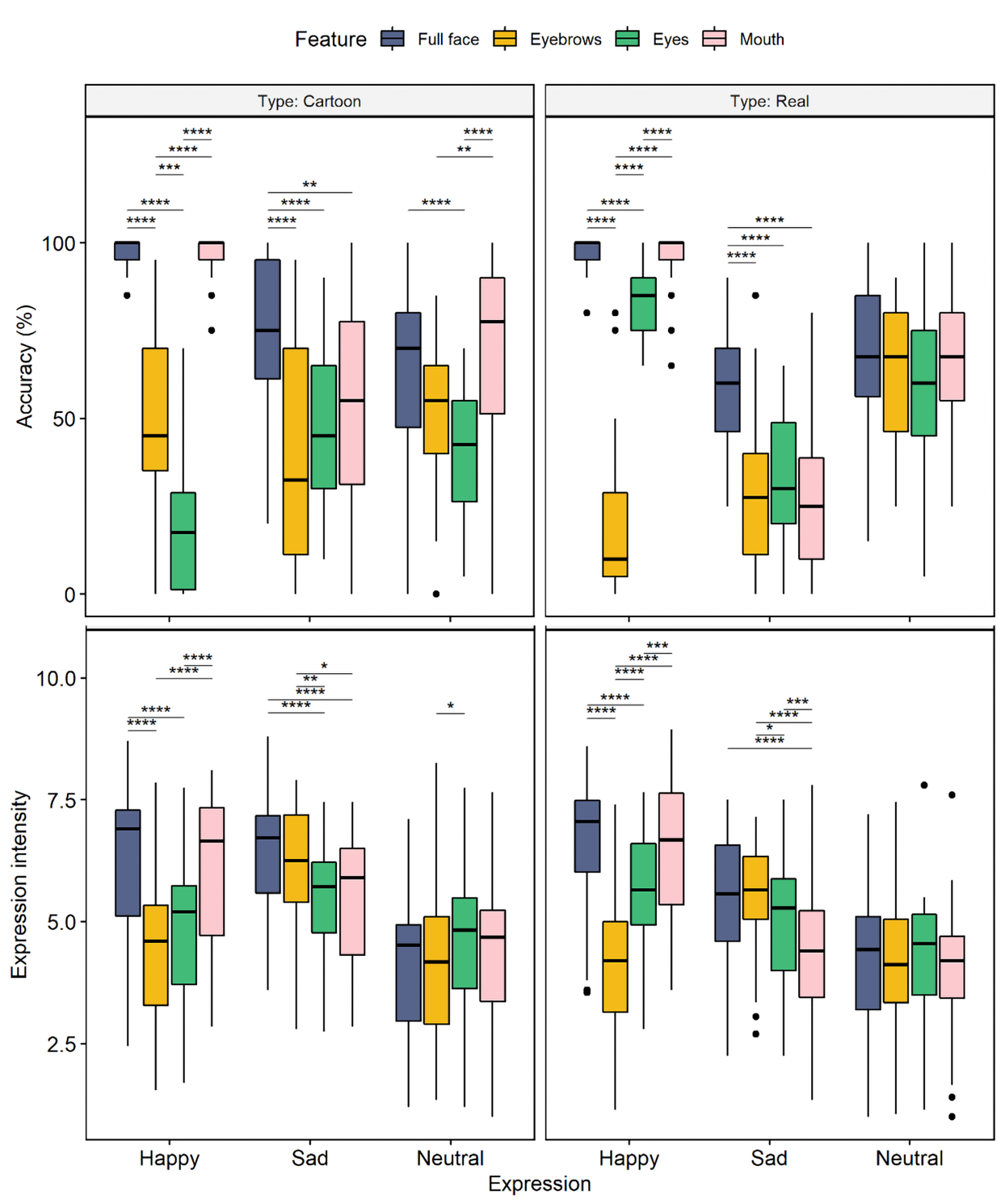
**

**Supplementary Figure 1.** Box plots for recognition accuracy (top) and perceived expression intensity (bottom) for the assessment of the expressions in cartoon (left) and real (right) faces as a function of facial features in Experiment 2. Note: Here and henceforth stars indicate the following levels of significance: **p* < .05, ***p* < .01, ****p* < .001, *****p* < .0001, in multiple contrasts, after Bonferroni corrections.


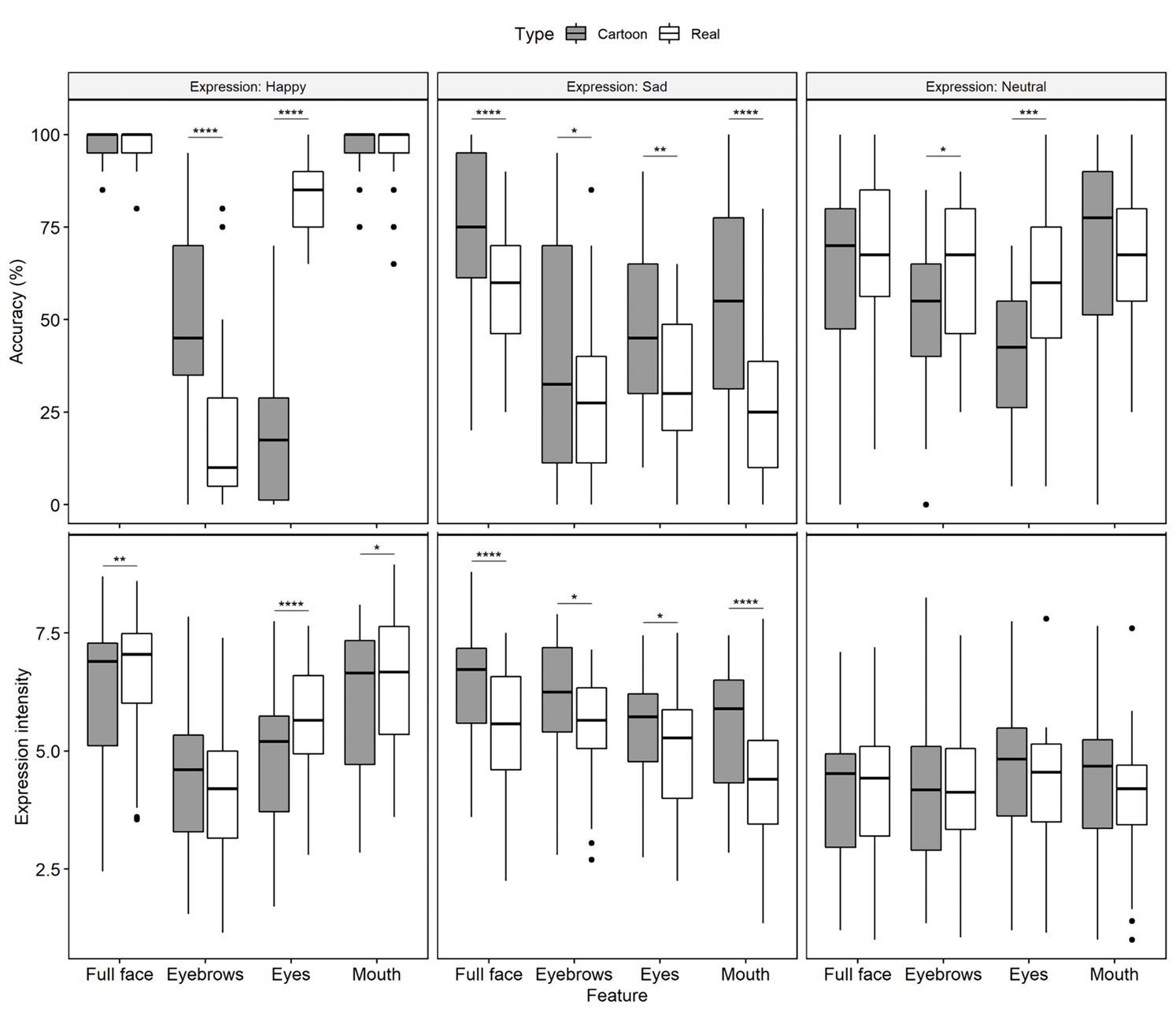


**Supplementary Figure 2.** Box plots for recognition accuracy (top) and perceived expression intensity (bottom) for judging cartoon and real faces expressions as a function of facial features in Experiment 2.


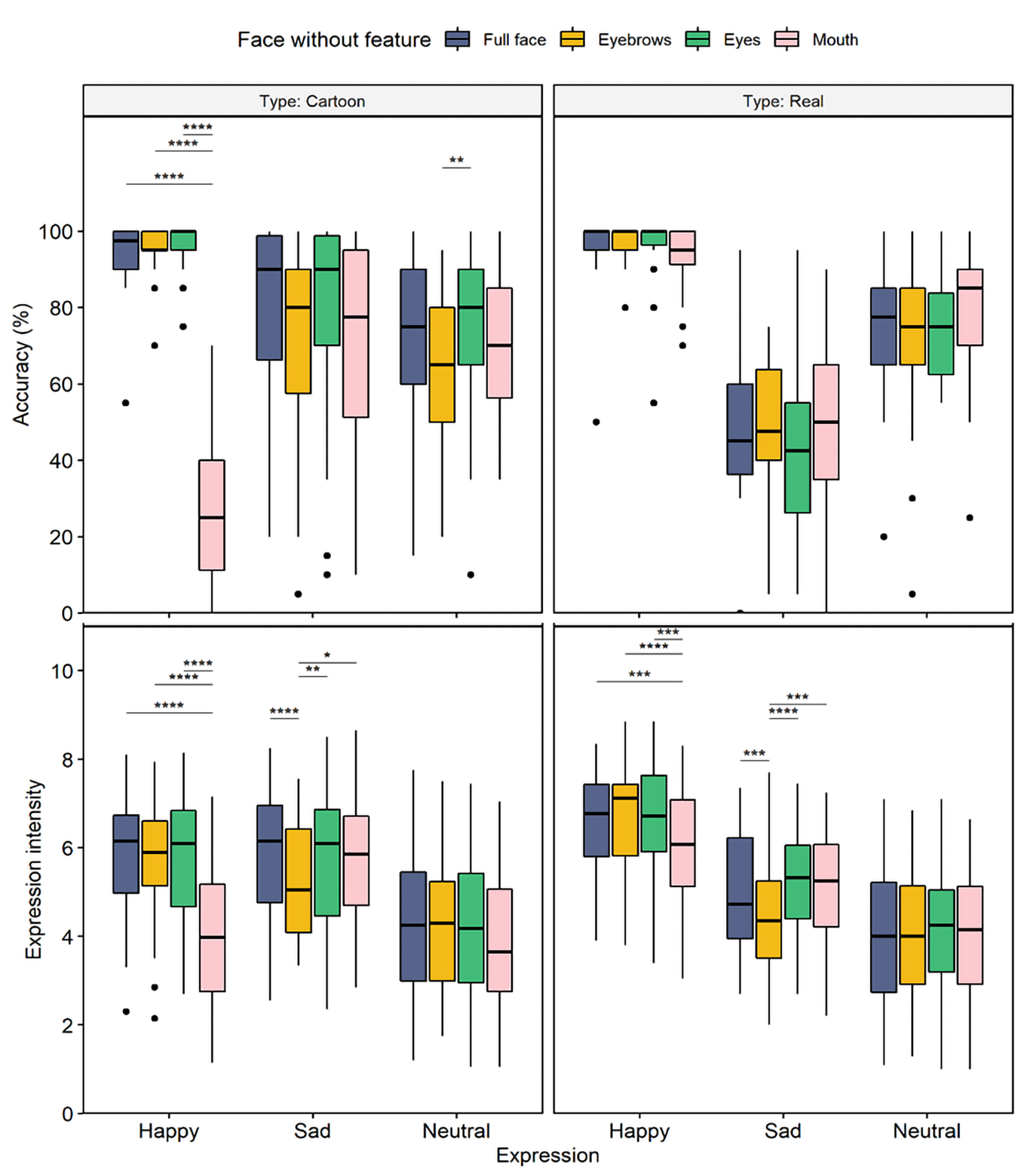


**Supplementary Figure 3.** Box plots for recognition accuracy (top) and perceived expression intensity (bottom) for the assessment of the expressions in cartoon (left) and real (right) faces as a function of faces without features in Experiment 3.


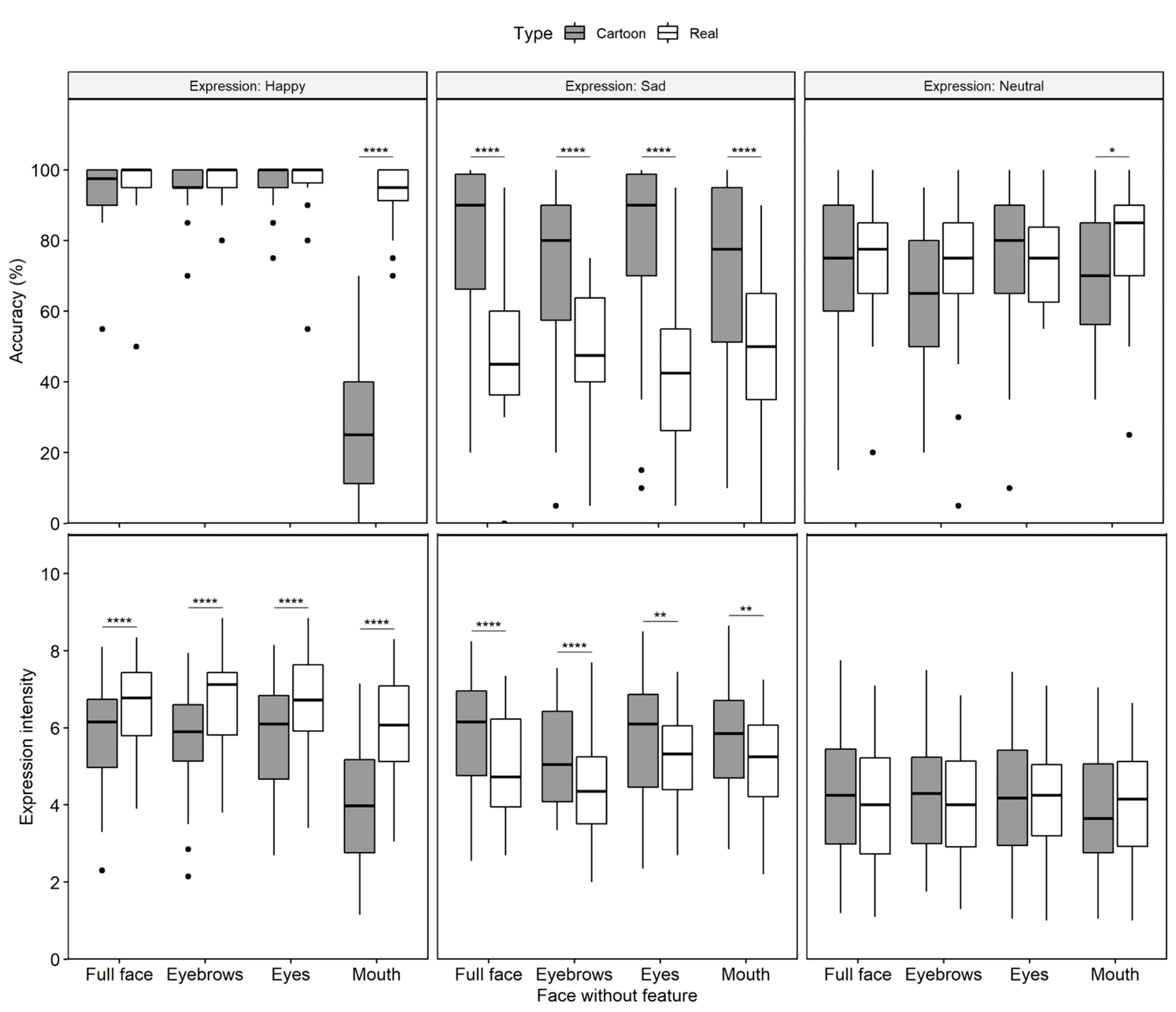


**Supplementary Figure 4.** Box plots for recognition accuracy (top) and perceived expression intensity (bottom) for judging cartoon and real faces expressions as as a function of faces without features in Experiment 3.
